# Untargeted and Targeted Metabolomics Reveal the Underlying Mechanism of Aspirin Eugenol Ester Ameliorating Rat Hyperlipidemia *via* Inhibiting FXR to Induce CYP7A1

**DOI:** 10.3389/fphar.2021.733789

**Published:** 2021-11-25

**Authors:** Lu Xiao-Rong, Ma Ning, Liu Xi-Wang, Li Shi-Hong, Qin Zhe, Bai Li-Xia, Yang Ya-Jun, Li Jian-Yong

**Affiliations:** ^1^ Key Lab of New Animal Drug Project of Gansu Province, Key Lab of Veterinary Pharmaceutical Development of Ministry of Agriculture and Rural Affairs, Lanzhou Institute of Husbandry and Pharmaceutical Science of Chinese Academy of Agricultural Sciences, Lanzhou, China; ^2^ College of Veterinary Medicine, Hebei Agricultural University, Baoding, China

**Keywords:** aspirin eugenol ester, hyperlipidemia, metabolomics, bile acids, cholesterol

## Abstract

Hyperlipidemia is an important lipid disorder and a risk factor for health. Aspirin eugenol ester (AEE) is a novel synthetic compound which is made up of two chemical structural units from aspirin and eugenol. Therapeutic effect of AEE on hyperlipidemia has been confirmed in animal model. But the action mechanism of AEE on hyperlipidemia is still poorly understood. In this study, we investigated the effects of AEE on liver and feces metabolic profile through UPLC-Q-TOF/MS-based untargeted metabolomics in hyperlipidemia hamster induced with high fat diet (HFD), and the effects of AEE on the expression of genes and proteins related to cholesterol and bile acid (BA) in HFD-induced hyperlipidemia SD rat. The concentrations of 26 bile acids (BAs) in the liver from hyperlipidemia SD rat were also quantified with the application of BA targeted metabolomics. The results of untargeted metabolomics showed that the underlying mechanism of AEE on hyperlipidemia was mainly associated with amino acid metabolism, glutathione metabolism, energy metabolism, BA metabolism, and glycerophospholipid metabolism. AEE induced the expression of the BA-synthetic enzymes cholesterol 7α-hydroxylase (CYP7A1) by the inhibition of BA nuclear receptor farnesoid X receptor (FXR) in liver, which resulted in accelerating the conversion of cholesterol into bile acids and excrete in feces. The results of BA targeted metabolomics showed that AEE elevated the glycine-conjugated BA level and decreased the tauro-conjugated BA level. In conclusion, this study found that AEE decreased FXR and increased CYP7A1 in the liver, which might be the possible molecular mechanisms and targets of AEE for anti-hyperlipidemia therapies.

## Introduction

As a multi-factorial disease, hyperlipidemia has been becoming a non-negligible health problem and is considered to be one of the main causes of cardiovascular disease (CVD) (Bonora, 2006; Prasun, 2020). CVD is a leading cause of death, and the blood lipids play a critical role in diagnosis and treatment of hyperlipidemia and CVD (Karr, 2017). Serum lipids mainly include triglycerides (TG), total cholesterol (TCH), low density lipoprotein cholesterol (LDL), and high density lipoprotein cholesterol (HDL), and the disorder of serum lipids causes the development of hyperlipidemia (Wasan et al., 1997; Li et al., 2016a). Hence, the accumulation of serum lipids in blood stream and liver is the main cause of hyperlipidemia, and the recovery of serum lipids is the leading part in hyperlipidemia treatment.

Metabolic disorder of bile acid (BA) and cholesterol is the main cause of hyperlipidemia. Liver is the main organ responsible for cholesterol synthesis and BAs conversion. Synthesis and excretion of BA play important roles in cholesterol and lipid metabolism, which are closely related to hyperlipidemia (Cerqueira et al., 2016). The classical or neutral pathway and alternative or acidic pathway are two pathways that cholesterol is converted to BA. The main production of the classical or neutral pathway catalyzed by CYP7A1 and oxysterol 12 α-hydroxylase (CYP8B1) is cholic acid (CA). The alternative acidic pathway is catalyzed by sterol-27α hydroxylase (CYP27A1) and oxysterol 7α-hydroxylase (CYP7B1), and the main production is chenodeoxycholic acid (CDCA) (Thomas et al., 2008; Chiang, 2009; Chambers et al., 2019). The BA is induced into the gallbladder and then into the intestines under the stimulation of feeding. The BAs can combine with glycine and taurine to form conjugated BA such as glycine-conjugated BAs (G-BAs) and taurine-conjugated BAs (T-BAs) (Schwarz et al., 1996), and most of BAs return to liver from the portal vein and a part of them is excreted in feces (Li-Hawkins et al., 2002; Chiang, 2009).

Bile acid metabolism is regulated by a multitude of factors, including BA receptors, transporters, genes, and proteins related to BAs that enter ohepatic circulation. Farnesoid X receptor (FXR) is a key receptor to regulate the homeostasis of BAs, and CDCA is the most effective ligand for FXR (Watanabe et al., 2004; Thomas et al., 2008). Many studies showed that FXR inhibited CYP7A1 a rate-limiting enzyme of hepatic BA synthesis (Watanabe et al., 2004; Thomas et al., 2008). BA homeostasis is also regulated by BA transporters. The export of BA from liver is regulated by bile salt export pump (BSEP), which is the rate limiting step in the progress of BA transport, and facilitates the BA enterohepatic circulation (Kullak-Ublick et al., 2004). Hepatocytes reabsorb the BA *via* the sodium taurocholate cotransporting polypeptide (NTCP) (Stieger, 2011). Bile acids are converted from liver cholesterol, so cholesterol metabolism plays pivotal roles in maintaining BA homeostasis. Sterol-regulatory element-binding protein 1c (SREBP1c) promotes fatty acid synthesis, and ATP-binding cassette transporter A1 (ABCA1) drives cholesterol efflux (Calkin and Tontonoz, 2012). Multidrug resistance-associated protein 2 (MRP2) is the main transport of bilirubin, which is responsible for the excretion of bilirubin and other bile components (Keppler, 2014).

Increasing literatures have reported that aspirin and eugenol have therapeutic effects on hyperlipidemia. Aspirin could improve endothelial dysfunction and reduce progression of atherosclerosis in mice (Yamamoto et al., 2010). Previous studies demonstrated the anti-hyperlipidemia potential of eugenol in experimental animal models, which may be due to the anti-oxidative properties and lipid-lowing effects (Venkadeswaran et al., 2016). However, the gastrointestinal damage caused by aspirin and chemical instability of eugenol limit their clinical applications. Carboxyl group of aspirin and hydroxyl group of eugenol are responsible for these limitations. In order to reduce the disadvantages and increase therapeutic effects, based on pro-drug principle, aspirin eugenol ester (AEE) was synthesized to mask the carboxyl group and hydroxyl group (Li et al. 2012). AEE, a white and odorless crystal, is a safe compound with good druggability and reduced toxicity. Metabolism study indicated that AEE would be decomposed into salicylic acid and eugenol after administration, and then its metabolites could show their original activities and act synergistically (Shen et al., 2015). Our previous studies also suggested that AEE was a promising drug candidate for normalizing blood lipid levels and displayed cholesterol-lowering properties in hyperlipidemic rats, but the underlying mechanism has not been elucidated (Karam et al., 2015; Karam et al., 2016).

Metabolomics is a versatile tool in comprehensively understanding of molecular mechanism and the response of metabolic pathways to perturbation. As a discipline in system biology, metabolomics has been extensively applied in investigating the altered metabolites of normal, pathological, or drug-treated subjects, which could provide important information for the therapeutic effect mechanism (Fillet and Frederich, 2015). As a fast metabolomic analysis technique, ultra-performance liquid chromatography-quadrupole time-of-flight mass spectrometry (UPLC-Q-TOF/MS) is considered to be an adequate platform for metabolomic study (Zhang et al., 2016). As important biological samples, feces and liver tissue are attractive for biomarker investigation to provide a new insight into the progression of hyperlipidemia and the therapeutic basis of AEE. As a highly sensitive and accurate method, liquid chromatography mass spectrometer (LC-MS/MS) is used to quantify the BA concentration of liver, plasma, bile, and urine (Alnouti et al., 2008; Zhang et al., 2020).

In this study, we investigated the underlying mechanism of AEE on hyperlipidemia. Untargeted metabolomics approach by performing UPLC-Q-TOF/MS analysis was employed to investigate the effects of AEE on fecal and hepatic metabolomics profiles in hyperlipidemia hamster. In order to gain further insight into the potential mechanism of AEE on hyperlipidemia, we also studied the expression of genes and proteins related to cholesterol and BA metabolism in hyperlipidemia SD rat. Furthermore, targeted metabolomic analysis was utilized to quantify the concentrations of 26 kinds of BAs in SD rat liver. To sum up, the study provided novel evidence in support of AEE improving hyperlipidemia, and showed that AEE was a potential drug for the treatment of hyperlipidemia.

## Materials and Methods

### Chemicals and Reagents

Transparent crystal AEE with purity of 99.5% was prepared in Key Lab of New Animal Drug Project of Gansu Province, Key Lab of Veterinary Pharmaceutical Development of Ministry of Agriculture and Rural Affairs, Lanzhou Institute of Husbandry and Pharmaceutical Science of Chinese Academy of Agricultural Sciences. MS-grade formic acid was supplied by TCI (Shanghai, China). Deionized water (18 MΩ) was prepared with a Direct-Q^®^3 system (Millipore, United States). MS-grade acetonitrile was purchased from Thermo Fisher Scientific (United States). Carboxymethylcellulose sodium (CMC-Na) was supplied by Tianjin Chemical Reagent Company (Tianjin, China). Normal diet (12.3% lipids, 63.3% carbohydrates, and 24.4% proteins) was purchased from Keao Xieli Feed Co., Ltd. (Beijing, China), and the high fat diet (HFD) (40% lipids, 43% carbohydrates, and 17% proteins) was supplied by Research Diet, Inc. (product D12079B, New Brunswick, NJ). The TG, TCH, LDL, and HDL kits for serum were provided by Ningbo Medical System Biotechnology Co., Ltd. (Ningbo, China). Erba XL-640 analyzer (German) was used to measure blood lipid levels. The total bile acid (TBA) kit for feces was provided by Nanjing Jiancheng Bioengineering Institute (Nanjing, China). The BA standards were purchased from Sigma-Aldrich and Steraloids.

### Fecal and Hepatic Untargeted Metabolomics Analysis in Hyperlipidemia Hamster

#### Animal Experiment and Study Design

Thirty male Syrian golden hamsters aged 11 weeks with weight of 100–110 g were purchased from Charles River Company (Vital River, Beijing, China). Hamsters had free access to their food and water. All animals were housed in facilities by group at 22 ± 2°C, with controlled relative humidity (45–65%). Hamsters were assigned into three groups (*n* = 10): 1) control group, in which hamsters were fed with normal diet; 2) high fat diet (HFD) group, in which hamsters were fed with HFD; 3) AEE group, in which hamsters were simultaneously fed with HFD and AEE (27 mg/kg body weight). AEE suspensions were prepared in 0.5% CMC-Na, and hamsters in AEE group were intragastrically (i.g.) administered with AEE. For eliminating the effect of CMC-Na (vehicle), hamsters in control and HFD groups were treated with equal volume of CMC-Na as AEE group. The experimental duration was 12 weeks until sacrifice. The untargeted metabolomics workflow was shown in Supplementary Figure S1. All experimental protocols and procedures were approved by the Institutional Animal Care and Use Committee of Lanzhou Institute of Husbandry and Pharmaceutical Science of Chinese Academy of Agricultural Sciences (Approval No. NKMYD202005; Approval Date: October 18, 2020). Animal welfare and experimental procedures were performed strictly in accordance with the Guidelines for the Care and Use of Laboratory Animals issued by the United States National Institutes of Health.

#### Sample Collection and Preparation

All the hamsters were survived during the duration of the experiment. Individual hamsters were placed in metabolic cages (1 per cage) to obtain 24-h fecal collections, and fecal samples were stored at −80°C before analysis. At the end of the experiment, hamsters were anesthetized using sodium pentobarbital by intraperitoneal injection at the dosage of 30 mg/kg body weight. The liver samples were carefully isolated, immediately snap frozen using liquid nitrogen, and then stored at −80°C for metabolomic analysis.

In order to ensure equal dry weight, fecal samples were lyophilized and pulverized (Sinha et al. 2016). Weighed fecal samples were mixed with methanol to extract metabolites as described previously (Cao et al. 2011). Briefly, 0.2 g fecal samples were placed into EP tubes, and 600 μl methanol was added. The mixture was swirled for 60 s, extracted by ultrasound for 10 min, and then centrifuged (13,000 rpm, 10 min, 4°C). The supernatants were collected and filtered by 0.22 μm nylon filter. An aliquot of 2 μl was injected for analysis. Prior to analysis, liver tissues were thawed at room temperature, and then 0.2 g liver tissue was weighed and mixed with 2 ml methanol/water (4:1, v:v, −20°C), swirled for 1 min, and homogenated to extract the compounds from liver. Then, the homogenate was further ultrasonically broken for 8 min with a frequency of 28 kHz (KQ-600DE, Kunshan Ultrasonic Instruments Co. Ltd., Kunshan, China) and incubated for 10 min in an ice bath. The mixture was centrifuged (15,000 rpm, 15 min, 4°C) to precipitate the proteins, and 1.6 ml of the supernatant was evaporated with a vacuum dryer. Finally, the residue was reconstituted with 200 μl methanol/water (4:1), and an aliquot of 3 μl was injected for UPLC–Q-TOF/MS analysis (Huang et al. 2011).

#### UPLC-MS Conditions in Untargeted Metabolomics

Metabolomics analysis was performed on an Agilent 1290 UPLC system coupled with an Agilent 6530 Q-TOF mass spectrometer. Chromatographic separations of fecal and liver samples were performed on an Agilent ZORBAX Eclipse plus C18 RRHD column (2.1 × 150 mm, 1.8 μm) maintained at 35°C. Ultrapure water with 0.1% formic acid (A) and acetonitrile with 0.1% formic acid (B) constituted the mobile phase, and the optimized gradient elution program for fecal and liver samples is shown in Supplementary Table S1. The post time was set to 5 min for equilibration. Mass spectrometry was performed both in electrospray ionization in positive (ESI+) and negative (ESI−) ion modes. The source parameters were set as follows: drying gas flow (nitrogen), 10 L/min at 350°C; capillary voltages of 4.0 KV in ESI+ and 3.5 KV in ESI−; fragment voltage, 135 V; skimmer voltage, 65 V; the nebulizer pressure, 45 psig; acquisition rate 1 spectra/s. Data was collected in centroid mode from 50–1,000 m/z using an extended dynamic model.

#### Data Processing and Statistical Analysis in Untargeted Metabolomics

The raw MS data were firstly exported by Mass Hunter Qualitative Analysis software (Version B6.0, Agilent technologies, United States) to converted to common data format (mzData). The program XCMS was used for nonlinear alignment of the data in the time domain and automatic integration and extraction of the peak intensities, with the default parameter settings. The data were filtered by interquantile range and normalized to the total intensity for further multivariate data analysis by MetaboAnalyst. SIMCA-P was used to performed data set analysis (version 13.0, Umetrics AB, Sweden). Principal component analysis (PCA) and partial least squares discriminant analysis (PLS-DA) were performed to identify the important variables with discriminative power. PLS-DA models were described by R^2^X, R^2^Y, and Q^2^, and its validity was evaluated by permutation testing (with 200 permutations). Variable importance in the projection (VIP > 1) value from PLS-DA model and the *p* values of one-way ANOVA (*p* < 0.05) were taken as the measurement indices for potential metabolites selecting.

#### Metabolites Identification and Pathway Analysis

TOF-MS accurate mass value of the metabolites of interest was searched against the METLIN or Human Metabolome Database (HMDB). Tandem mass spectrometry (MS/MS) analysis was carried out to confirm the structure of potential biomarkers by matching the masses of the fragments. Pathway and heatmap analysis were performed on MetaboAnalyst 3.0 (http://www.metaboanalyst.ca/), and a literature search was conducted to identify the affected metabolic pathways and to facilitate further biological interpretation.

### Analysis of Genes and Proteins Related to BA and Cholesterol Metabolism

#### Animals and Treatments

Sprague-Dawley male rats aged 5 weeks old and weighted 100–120 g were purchased from Lanzhou University (Lanzhou, China). The rats were raised in living conditions with a 12 h light/dark cycle at 18–22°C and 50 ± 10% humidity, and acclimated for a week before the study beginning. The protocols and procedures for this animal study were approved by the Institutional Animal Care and Use Committee of Lanzhou Institute of Husbandry and Pharmaceutical Science of Chinese Academy of Agricultural Sciences (Approval No. NKMYD201905; Approval Date: March 18, 2019). Animal welfare and experimental procedures were performed strictly in accordance with the Guidelines for the Care and Use of Laboratory Animals issued by the United States National Institutes of Health.

Forty rats were randomly divided into two groups: the control group, in which rats were received normal diet (ND) for 8 weeks (*n* = 20), and HFD group, in which rats were received HFD for 8 weeks (*n* = 20). After 8 weeks, 10 rats in ND group were randomly to be chosen in ND and AEE group (ND + AEE), in which rats were administrated with AEE (54 mg/kg body weight) and normal diet simultaneously. After hyperlipidemia established successfully, 10 rats in HFD group were randomly to be chosen in HFD and AEE treatment group (HFD + AEE), in which AEE was given by gavage with HDF simultaneously. The dosage of AEE was 54 mg/kg per day, and the administration period was lasted for 5 weeks (Karam et al., 2016). AEE suspensions were prepared in 0.5% CMC-Na, and the rats in normal and HFD groups were received equal volume of 0.5% CMC-Na as AEE treatment group.

#### Analysis of Total BAs, Blood Lipids, and Histological Examination

At the end of the experiment, fresh feces were collected by gently touching the lower abdomen of rats and then dried with freeze-drying apparatus. The feces were stored at −20°C to detect the TBA content of feces by multifunctional enzyme mark. Rats were fasted for 10–12 h before being euthanized by injecting pentobarbital sodium (30 mg/kg body weight). Blood samples were collected from the heart into heparin-treated vacuum tubes, and plasma samples were obtained through centrifugation (3,500 rpm for 10 min at 4°C) and then stored at −80°C for blood lipids analysis. Liver was carefully collected and kept in liquid nitrogen and stored at −80°C. For histological examination of the liver, the liver tissue was fixed with 10% formaldehyde, embedded in paraffin wax, sectioned to a thickness of 5 µm, and stained with hematoxylin-eosin staining (H&E) (Supplementary Figure S2). Finally, the slides were examined under a light microscope.

#### Gene Expression Analysis

Real-time quantitative PCR (RT-qPCR) was employed to study the effects of AEE on gene expression involved in cholesterol and bile acid metabolism. Total RNA was extracted from liver tissue using a RNeasy mini kit (TaKaRa, MiniBEST Universal RNA Extraction kit, Code No.9767), and reverse transcription reaction was conducted using a Superscript kit (TaKaRa, PrimeScriptTMRT reagent Kit with gDNA Eraser, Code No. RR036A) following the manufacturer’s instruction. The quality of RNA was checked by spectrophotometry. RT-qPCR was performed using a Power SYBR Green PCR Master Mix kit (TaKaRa, TB GreenTM Premix Ex TaqTM, Code No. RR820A) on an ABI ViiATM7 system. The expression level of target gene mRNA was normalized to the mRNA level of glyceraldehyde 3-phosphate dehydrogenase. The relative abundance of the target gene expression was calculated by the 2−^△△CT^ method. The sequences of primers used are listed in Supplementary Table S2.

#### Protein Expression Analysis

Total protein was extracted from liver using cold RIPA lysis buffer. Protein were separated on a precast SDS-PAGE gel (4–20%). The isolated proteins were transferred to a polyvinylidene fluoride (PVDF) membrane. Blots were incubated with the primary antibody followed by horseradish peroxidase-conjugated secondary antibody. Results were detected using the G: Box Chemi XRQ Imaging System (Cambridge, United Kingdom). Antibodies used in this study were listed in Supplementary Table S3.

### Targeted Metabolomics Analysis of Hepatic BAs

The SD rat liver tissue (30 mg) was homogenized in 300 µl water, and then 100 µl homogenate was diluted with 900 µl ultrapure water. Diluted homogenate (100 µl) was taken and mixed with 500 µl of prechilled methanol and 10 µl of inner standard (IS), and then the mixture was incubated at −20°C for 20 min to precipitate the protein. After that, the mixture was centrifuged at 12,000 rpm for 15 min (4°C) to gain the supernatant, from which 400 µl of supernatant was taken, evaporated under vacuum, and reconstituted in 100 µl methanol-water (1:1, V/V). The suspension was centrifuged at 12,000 rpm for 15 min (4°C), and then the supernatant was ready for injection. In rat liver, the concentrations of 26 BAs including cholic acid (CA), chenodeoxycholic acid (CDCA), deoxycholic acid (DCA), ursodeoxycholic acid (UDCA), hyodeoxycholic acid (HDCA), glycocholic acid (GCA), glycochenodeoxycholic acid (GCDCA), glycodeoxycholic acid (GDCA), glycoursodeoxycholic acid (GUDCA), glycohyodeoxycholic acid (GHDCA), taurocodeoxycholic acid (TDCA), taurochenodeoxycholic acid (TCDCA), tauroursodeoxycholic acid (TUDCA), taurohyodeoxycholic acid (THDCA), glycolithocholic acid (GLCA), lithocholic acid (LCA), taurocholic acid (TCA), taurolithocholic acid (TLCA), α-muricholic acid (α-MCA), β-muricholic acid (β-MCA), apocholic acid (ApoCA), 3-dehydrocholic acid/3-oxocholic acid (3-DHCA), 7-ketodeoxycholic acid (7-KDCA), murocholic acid (MoCA), isolithocholic acid (IsoLCA), and allocholic acid (AlloCA) were quantified by LC-MS/MS. Separation of BAs was carried out on Agilent 1,290 Infinity LC system equipped with ACQUITY BEH-C18 threaded column (2.1 × 100 mm, 1.7 µm). The sample was placed in an automatic sampler at 8°C, the column was maintained at 45°C, the flow rate was 250 µl/min, and the sample was injected at 2 µl. Mobile phase A was water with 0.1% formic acid, while mobile phase B was methanol. The gradient program was optimized as follows: 0–7 min 60–70% B; 7–15 min 70–85% B; 15–17 min 85% B; 17–17.1 min 85–60% B; 17.1–20 min 60% B. Mass spectrometry analysis was performed using 5500 QTRAP mass spectrometer (AB SCIEX) using multiple reaction monitoring mode in negative ion. The MS conditions were as follows: source temperature, 550°C; ion source gas1, 55 psi; ion source gas2, 55 psi; scan type; curtain gas, 40; ionsapary voltage floating, −4500V.

The standard curves of bile acids were provided in Supplementary Table S4. The peak area and retention time were extracted by Multiquant software. The retention time was corrected by bile acid standard, and the metabolites were identified.

### Statistical Analysis

All data are expressed as means ± SD. The statistical analysis was performed using SPSS software (version 19.0 SPSS). The differences among different treatment groups were analyzed by one-way ANOVA followed by a Dunnett post hoc test. *p*-values below 5% were considered significant.

## Results

### Liver Metabolic Profiling

**FIGURE 1:**
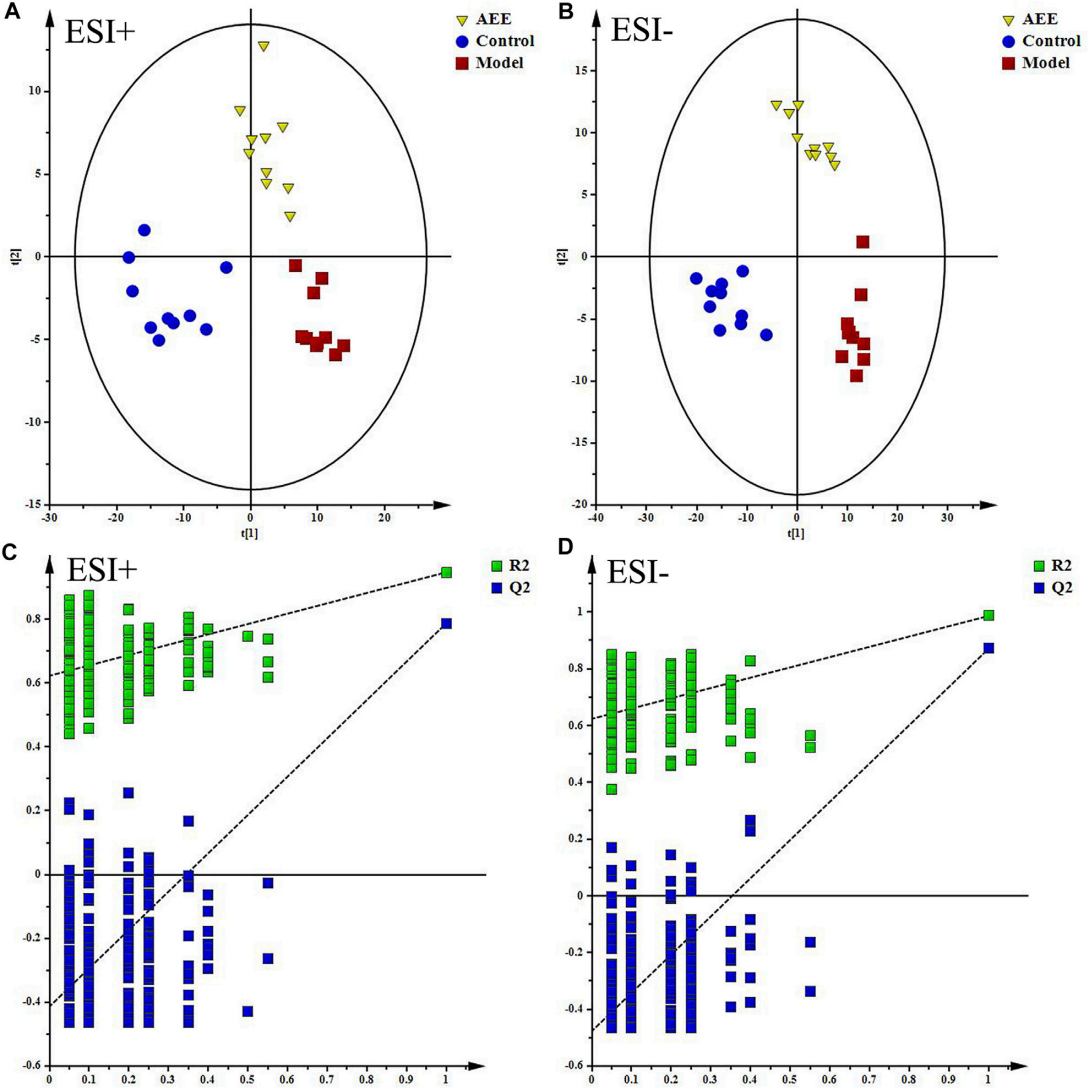
Effect of AEE on metabolomic profile of liver in hamster fed with high fat diet. **(A,B)** PLS-DA score plots of liver samples collected from different treatment groups, ESI+: R^2^X = 0.506, R^2^Y = 0.961, and Q^2^ = 0.838; ESI-: R^2^X = 0.535, R^2^Y = 0.982, and Q^2^ = 0.919. **(C,D)** Permutation test of PLS-DA models in ESI+ and ESI−.

With the application of UPLC-Q-TOF/MS analysis, the representative metabolic profiles of liver in ESI+ and ESI− were shown in Supplementary Figure S3. Total ion chromatograms (TICs) showed good separations and strong sensitivity of the optimal method. The datasets of the liver samples in positive and negative modes were provided in Supplementary File S1. PCA was applied to globally understand the metabolomic profile in all groups. PCA score plots in ESI+ and ESI− (Supplementary Figure S4) showed that the liver samples in model group were located away from those in the control implying that HFD had remarkable influence on liver metabolites. Meanwhile, the liver metabolic profile of hamster in AEE group differed from the model group. PLS-DA was also carried out to distinguish the differences between experimental groups. The score plots of the PLS-DA were depicted in Figure 1. A clear separation among control, model, and AEE groups was observed (Figures 1A,B). The metabolic profile of hamster in AEE group fairly differed from the model group, suggesting that the deviations induced by hyperlipidemia were significantly improved after AEE treatment. In order to guard against model overfitting, a default of seven rounds of cross-validation across was applied. Validation with 200 random permutation tests generated intercepts of R^2^ = 0.656 and Q^2^ = −0.393 in positive data and R^2^ = 0.624 and Q^2^ = −0.476 in negative data (Figures 1C,D), indicating the PLS-DA models with good predictive ability and reliability.

### Feces Metabolic Profiling

**FIGURE 2:**
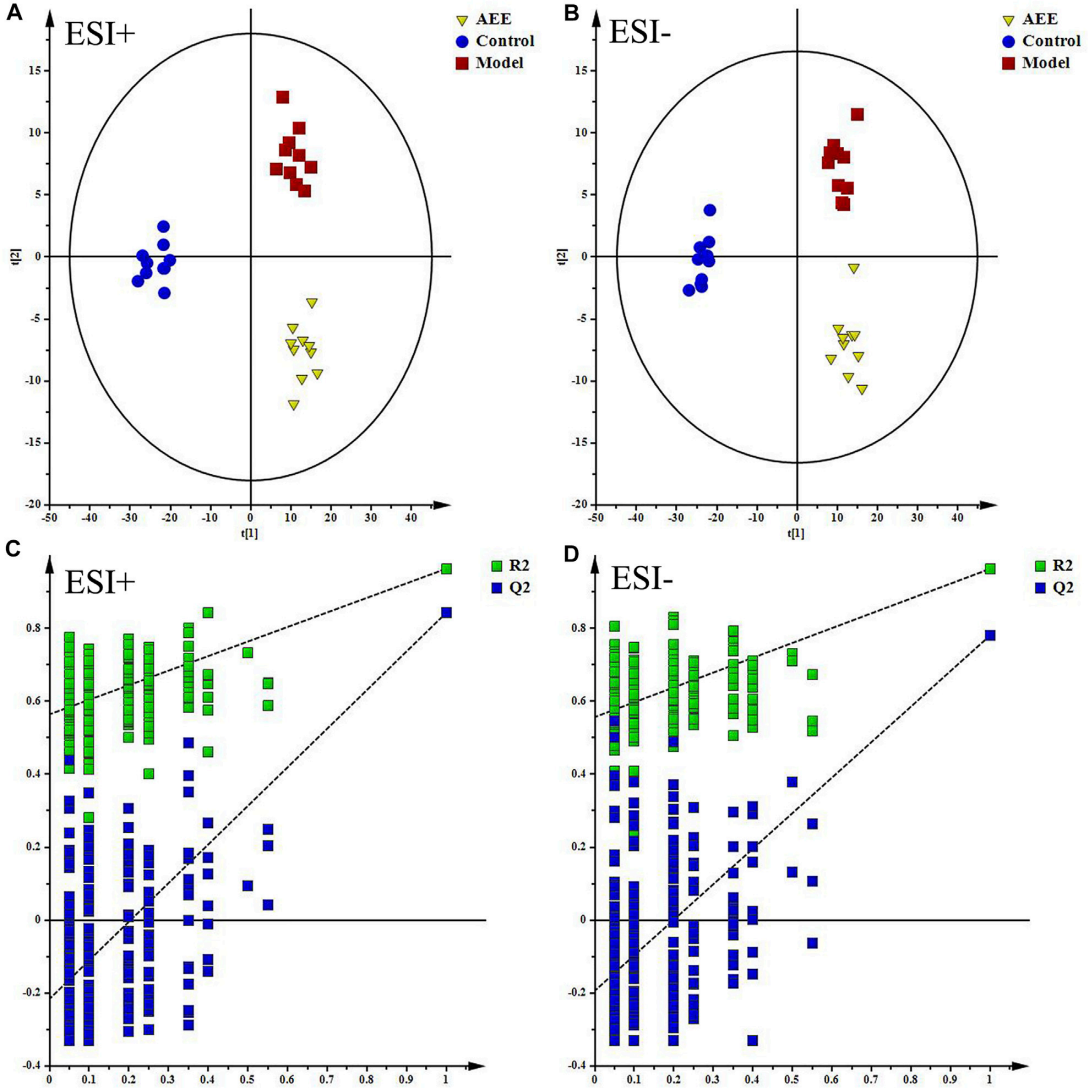
Effect of AEE on metabolomic profile of feces in hamster fed with high fed diet. **(A,B)** PLS-DA score plots of feces samples collected from different treatment groups, ESI+: R^2^X = 0.5, R^2^Y = 0.974, and Q^2^ = 0.889; ESI−: R^2^X = 0.524, R^2^Y = 0.973, and Q^2^ = 0.837. **(C,D)** Permutation test of PLS-DA models in ESI+ and ESI−.

Supplementary Figure S5 showed the typical TICs of fecal samples in positive and negative modes. The datasets of the fecal samples in positive and negative modes were provided in Supplementary File S2. PCA score plots of fecal samples indicated that the metabolomic profiles of hamster in control, model, and AEE groups were different, and the separations of three groups could be observed (Supplementary Figure S6). Detailed metabolomics differences among three groups were also revealed by PLS-DA models (Figures 2A,B). According to the score plots, the fecal samples in model group were located away from those in the control, indicating the success of hyperlipidemia model. AEE group clustered and deviated from model group, which suggested that AEE treatment partially improved the hyperlipidemic state. Results of the permutation test, intercepts of R^2^ = 0.564 and Q^2^ = −0.216 in positive data and R^2^ = 0.557 and Q^2^ = −0.193 in negative data, demonstrated that the original PLS-DA models were robust without overfitting (Figures 2C,D).

### Biomarker Identification and Cluster Analysis

With VIP values above 1 and *p*-values below 0.05, 24 metabolites in liver (Table 1) and 4 metabolites in feces (Table 2) were selected and identified as potential biomarker, which played significant roles in the formation of hyperlipidemia and contributed to the pharmacological action of AEE. Compared with the control group, biomarkers in the model group were significantly affected by HFD. Interestingly, the relative contents of the potential biomarkers were significantly reversed by AEE treatment (Tables 1, 2). In order to further understand the metabolic difference among groups, the relative content of biomarkers was displayed by heatmap in Supplementary Figure S6. It showed the metabolites with high (red) or low (blue) relative intensity, indicating that the metabolic patterns of the potential biomarkers were significantly disturbed in model group and AEE could improve these deviations. The sample and metabolite differences were also simultaneously hierarchically clustered to intuitively display their relationships. The vertical axil of Supplementary Figure S7 showed a dendrogram of the sample differences. Notably, samples in control and AEE groups were grouped closely, and samples in model group were branched separately, implying the positive treatment effects of AEE on hyperlipidemia. The dendrogram of the metabolite differences was shown in the horizontal axis in Supplementary Figure S7. Metabolites in same metabolic pathways or with similar change trend were firstly clustered together. For instance, Lysophosphatidylcholines (LysoPC) (18:0), LysoPC (16:1), and LysoPC (20:3) involved in glycerophospholipid metabolism were first clustered together; phenylalanine and tryptophan belonging to the amino acid metabolism pathway were also clustered together.

**TABLE 1:** Metabolites with significant changes in liver from hamster fed with high fed diet.

RT	SM	VIP	Formula	m/z	ME (ppm)	Metabolite	Pathway	Fold change	
								HFD/Control	AEE/HFD
1.3	+, −	2.73	C_10_H_17_N_3_O_6_S	308.0916	1.95	Glutathione	Glutathione metabolism	↑^**^	↓^**^
1.85	+	3.71	C_5_H_11_NO_2_S	150.0582	−0.67	Methionine	Cysteine and methionine metabolism	↓^**^	↑^**^
5.19	+	2.52	C_14_H_17_N_5_O_8_	384.1160	2.86	Succinyladenosine		↑^**^	↓^**^
18.49	+	4.83	C_26_H_54_NO_7_P	524.3726	3.05	LysoPC (18:0)	Glycerophospholipid metabolism	↑^**^	↓
1.30	+	2.35	C_5_H_11_NO_2_	118.0866	3.39	Betaine	Glycine, serine, and threonine metabolism	↓^*^	↑
1.57	+	1.80	C_9_H_8_O_3_	165.0546	−0.13	Phenylpyruvic acid	Phenylalanine metabolism	↓^*^	↑^*^
2.18	+	1.94	C_8_H_9_NO	136.0759	1.54	2-Phenylacetamide	Phenylalanine metabolism	↑^**^	↓^**^
14.76	+	2.41	C_24_H_48_NO_7_P	494.3259	3.62	LysoPC (16:1)	Glycerophospholipid metabolism	↑^**^	↓^*^
15.83	+	2.28	C_28_H_52_NO_7_P	546.3571	3.11	LysoPC (20:3)	Glycerophospholipid metabolism	↑^**^	↓^*^
4.49	+	1.12	C_5_H_11_NO_2_	118.0864	1.23	Valine	Valine, leucine and isoleucine metabolism	↓^*^	↑^*^
1.24	−	1.89	C_5_H_10_O_6_	165.0434	17.80	Ribonic acid	_	↓^**^	↑^**^
1.53	−	3.22	C_5_H_7_NO_3_	128.0377	18.70	Pyroglutamic acid	Glutathione metabolism	↓^**^	↑^**^
4.79	+, −	2.74	C_9_H_11_NO_2_	164.0743	15.85	Phenylalanine	Phenylalanine metabolism	↓^**^	↑^*^
24.60	−	4.27	C_18_H_36_O_2_	283.2677	12.36	Stearic acid	Biosynthesis of unsaturated fatty acids	↓^**^	↑^**^
1.31	−	1.54	C_4_H_6_O_5_	133.0168	19.20	Malic acid	Citrate cycle	↓^*^	↑^*^
2.32	+, −	1.68	C_9_H_11_NO_3_	180.0694	15.55	Tyrosine	Tyrosine metabolism	↓^*^	↑
5.78	−	1.51	C_11_H_12_N_2_O_2_	203.0865	19.20	Tryptophan	Tryptophan metabolism	↓^*^	↑
5.80	−	1.57	C_27_H_33_N_9_O_15_P_2_	784.1566	8.61	FAD	Riboflavin metabolism	↑^**^	↓^**^
10.88	−	1.53	C_26_H_43_NO_5_	448.3109	9.05	GUDCA	_	↑^**^	↓^**^
20.05	+, −	1.44	C_18_H_35_NO_3_	312.2581	11.82	Palmitoylglycine	_	↓^**^	↑^*^
20.61	−	1.49	C_20_H_37_NO_3_	338.2737	10.94	Oleoyl glycine	_	↓^**^	↑^**^
22.77	−	1.42	C_16_H_32_O_2_	255.2364	13.51	Palmitic acid	Fatty acid metabolism	↑^**^	↓^*^
1.44	−	1.34	C_5_H_4_N_4_O_2_	151.0288	17.76	Xanthine	Purine metabolism	↓^*^	↑^*^
1.85	−	1.10	C_4_H_4_N_2_O_2_	111.0224	21.62	Uracil	Pyrimidine metabolism	↓^**^	↑^**^

RT, retention time; VIP, variable importance in the projection; ME, mass error in ppm; SM, scan mode; FAD, flavin adenine dinucleotide; GUDCA, glycoursodeoxycholic acid; +, metabolites found in positive mode; −, metabolites found in negative mode; +, −, metabolites found in both positive and negative modes; _, Not available. Compared with the HFD group, *p < 0.05, **p < 0.01.

**TABLE 2:** Metabolites with significant changes in feces from hamster fed with high fed diet.

RT	SM	VIP	Formula	m/z	ME (ppm)	Metabolite	Pathway	Fold change	
								HFD/Control	AEE/HFD
10.82	+	1.51	C_18_H_39_NO_3_	318.3000	−0.85	Phytosphingosine	Sphingolipid metabolism	↑^**^	↓^**^
18.19	+	1.06	C_18_H_36_O_2_	285.2786	−0.70	Stearic acid	Fatty acid biosynthesis	↑^**^	↓
10.28	−	1.45	C_26_H_43_NO_6_	464.3032	3.10	Glycocholic acid	Bile metabolism	↑^**^	↑^**^
11.34	−	1.45	C_24_H_40_O_5_	407.2813	2.46	Cholic acid	Bile metabolism	↑	↑^*^

RT, retention time; VIP, variable importance in the projection; ME, mass error in ppm; SM, scan mode; +, metabolites found in positive mode; −, metabolites found in negative mode. Compared with the HFD group, ^*^ *p* < 0.05, ^**^ *p* < 0.01.

### Pathway Analysis of Untargeted Metabolomics

**FIGURE 3:**
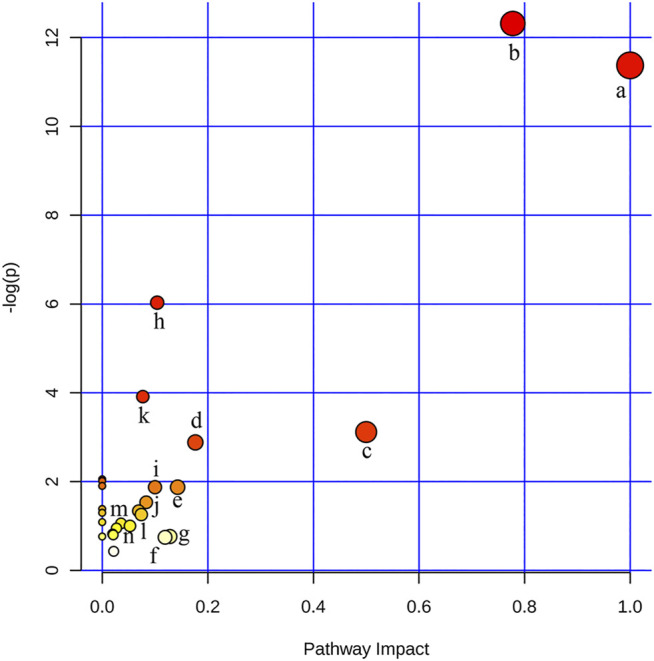
Disturbed pathways in response to hyperlipidemia and AEE treatment in hamster. The *x*-axis was the pathway impact value calculated from pathway topological analysis, and the *y*-axis was the -log (p) value obtained from pathway enrichment analysis. a, Phenylalanine, tyrosine, and tryptophan biosynthesis; b, Phenylalanine metabolism; c, Ubiquinone and other terpenoid-quinone biosynthesis; d, Glutathione metabolism; e, Valine, leucine, and isoleucine biosynthesis; f, Tryptophan metabolism; g, Tyrosine metabolism; h, Aminoacyl-tRNA biosynthesis; i, Riboflavin metabolism; j, Glyoxylate and dicarboxylate metabolism; k, Pantothenate and CoA biosynthesis; l, Pyruvate metabolism; m, Citrate cycle (TCA cycle); n, Glycerophospholipid metabolism.

In order to identify and visualize the affected metabolic pathways, metabolomics pathway analysis was performed with MetaboAnalyst. Supplementary Table S5 showed the summary of the pathway analysis. Figure 3 showed an overview of the pathway analysis with the most impacted pathways colored. The pathway with impact-value above 0.05 was filtered out. There were 14 disturbed pathways in response to hyperlipidemia and AEE treatment including phenylalanine, tyrosine and tryptophan biosynthesis, phenylalanine metabolism, ubiquinone and other terpenoid-quinone biosynthesis, glutathione metabolism, valine, leucine and isoleucine biosynthesis, tryptophan metabolism, tyrosine metabolism, aminoacyl-tRNA biosynthesis, riboflavin metabolism, glyoxylate and dicarboxylate metabolism, pantothenate and CoA biosynthesis, pyruvate metabolism, krebs cycle, and glycerophospholipid metabolism. These pathways were mainly classified into amino acid metabolism, glutathione metabolism, energy metabolism, and glycerophospholipid metabolism. Interestingly, the results of untargeted fecal metabolomics found that glycocholic acid and cholic acid were significantly increased by AEE treatment. Therefore, the effects of AEE on bile acid metabolism were further investigated.

### Effects of AEE on Blood Lipids, Liver Index, and TBA in Feces in Hyperlipidemia Rat

**FIGURE 4:**
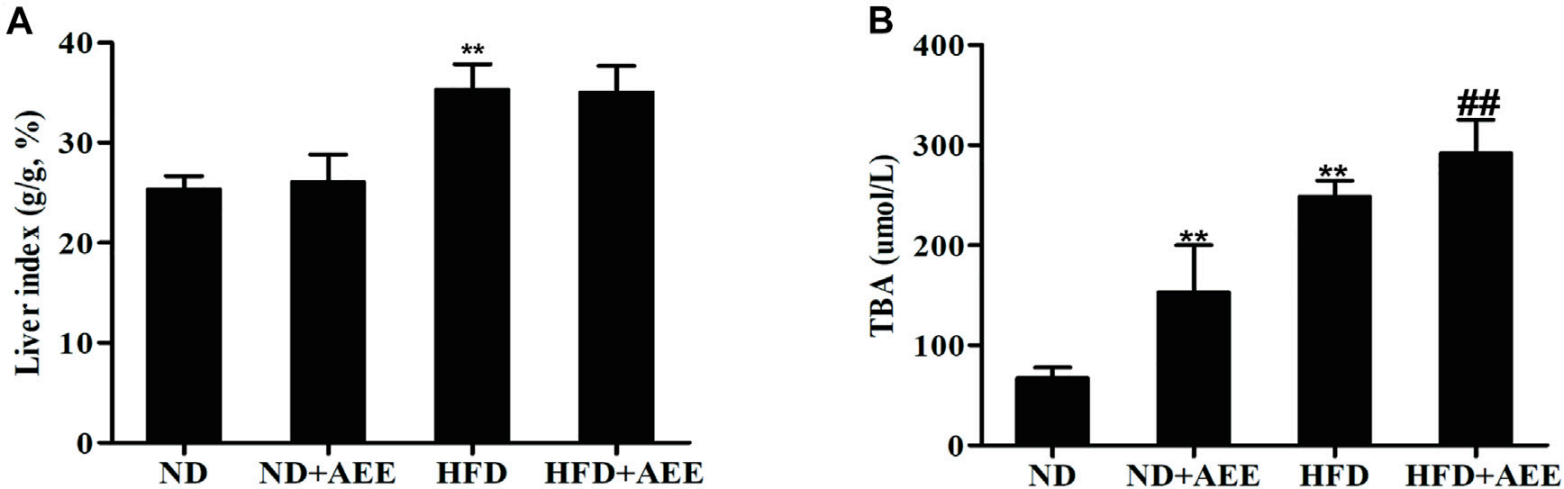
Effect of AEE on liver index and fecal TBA in hyperlipidemia rat. **(A,B)** Effect of AEE on liver index and fecal TBA in hyperlipidemia rat. **(A)** AEE reduced liver index in hyperlipidemia rat. Liver index: the ratio of liver weight to body weight (%). **(B)** The effect of AEE on TBA in feces of rats. Data are expressed as the means ± SD (*n* = 10). ND: Normal diet; HFD: high-fat diet; AEE: aspirin eugenol ester, ^**^ *p <* 0.01 compared with the ND group; ^##^ *p <* 0.01 compared with the HFD group.

The results of blood lipids were shown in Table 3. Levels of TCH, TG, and LDL were higher in the HFD group than those in the ND group (*p* < 0.01), whereas the HDL was significantly reduced (*p* < 0.01). In comparison with the ND group, TCH and LDL levels in ND + AEE group were significantly decreased (*p* < 0.01). Compared with HFD group, TCH, TG, and LDL levels of HFD + AEE group were significantly decreased (*p* < 0.01). As shown in Figure 4, in comparison with the ND group, the liver index induced by HFD increased significantly (*p* < 0.01). However, the liver index showed no significant difference between ND + AEE group and ND group, and also not statistically significant between HFD + AEE group and HFD group. Furthermore, fecal TBA excretion was increased significantly in ND + AEE group than in ND group, and also elevated significantly in HFD + AEE group than in HFD group (*p* < 0.01).

**TABLE 3:** Effect of AEE on blood lipids in rats with hyperlipidemia.

Variables	ND	ND + AEE	HFD	HFD + AEE
TCH (mmol/L)	1.27 ± 0.21	1.00 ± 0.16^**^	2.20 ± 0.23^**^	1.54 ± 0.21^##^
TG (mmol/L)	0.52 ± 0.13	0.47 ± 0.06	0.81 ± 0.19^**^	0.61 ± 0.15^##^
HDL (mmol/L)	0.58 ± 0.09	0.44 ± 0.08	0.38 ± 0.06^**^	0.36 ± 0.08
LDL (mmol/L)	0.34 ± 0.07	0.21 ± 0.05^**^	0.52 ± 0.07^**^	0.34 ± 0.06^##^

ND, normal diet; HFD: high-fat diet; AEE, aspirin eugenol ester; TCH, total cholesterol; TG, triglyceride; HDL, high density lipoprotein; LDL, low density lipoprotein. Data are expressed as the means ± SD (*n* = 10), ^**^ *p <* 0.01 compared with the ND group; ^##^ *p* < 0.01 compared with the HFD group.

### AEE Regulated Genes Related to Cholesterol and BA Metabolism

**FIGURE 5:**
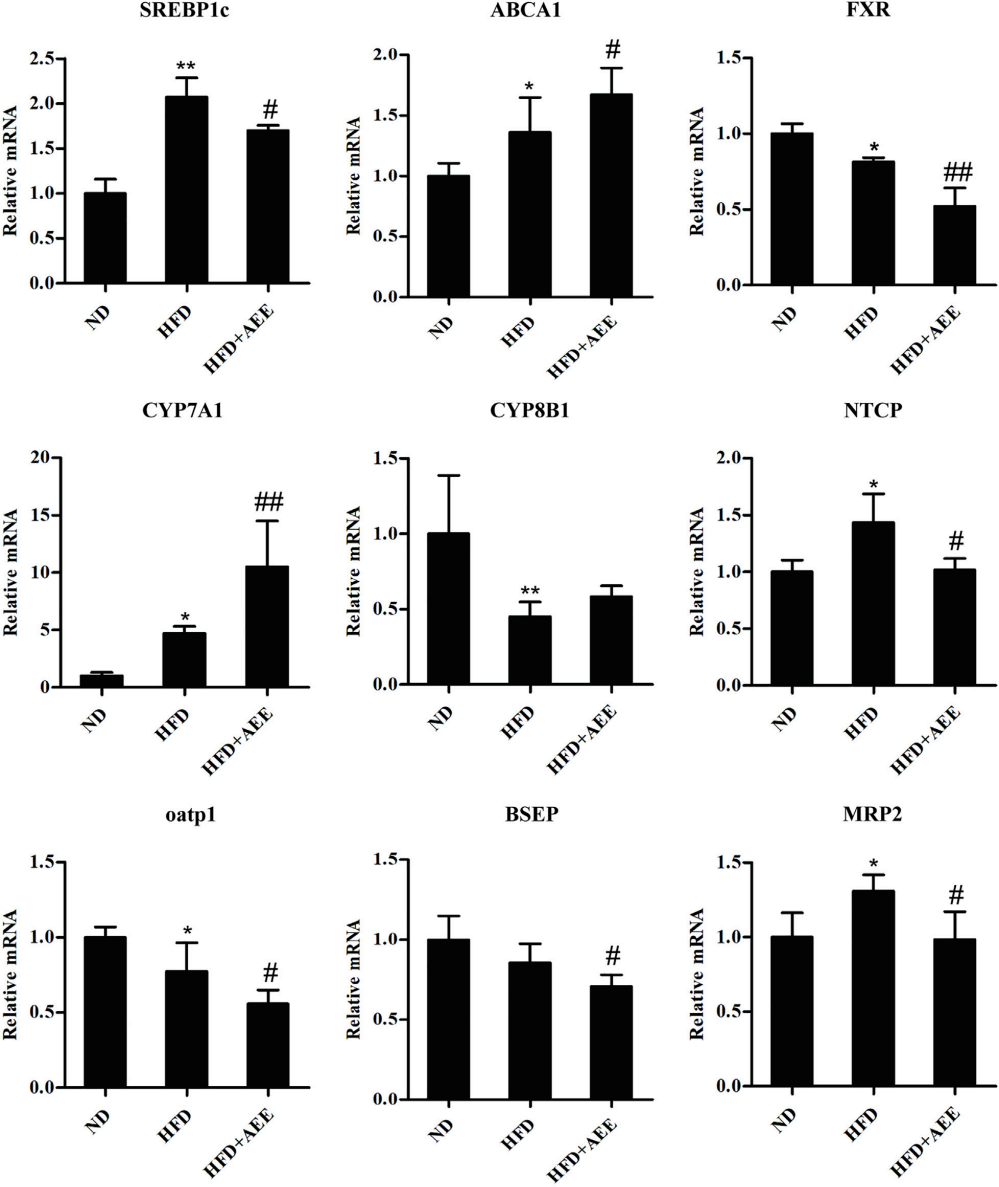
Effects of AEE on mRNA levels of genes related with cholesterol and BA metabolism in hyperlipidemia rat. Data are expressed as the means ± SD (*n* = 10). ND, control group; HFD, high-fat diet; AEE, aspirin eugenol ester. ^*^ *p* < 0.05, ^**^ *p* < 0.01 compared with the ND group; ^#^ *p* < 0.05, ^##^ *p* < 0.01 compared with the HFD group.

As shown in Figure 5, AEE effectively inhibited the expression of SREBP1c and promoted the expression of ABCA1. Figure 5 showed that AEE increased significantly the mRNA level of CYP7A1 (*p* < 0.01), and tended to increase CYP8B1 mRNA level but not reaching statistical significance. In addition, AEE significantly decreased the mRNA level of FXR in the liver in comparison with the HFD group (*p* < 0.01). Meanwhile, AEE decreased the BA enterohepatic circulation-related genes in the rat liver including oatp1, NTCP, BSEP, and MRP2 (*p* < 0.05).

### AEE Regulated Proteins Related to Cholesterol and BA Metabolism

**FIGURE 6:**
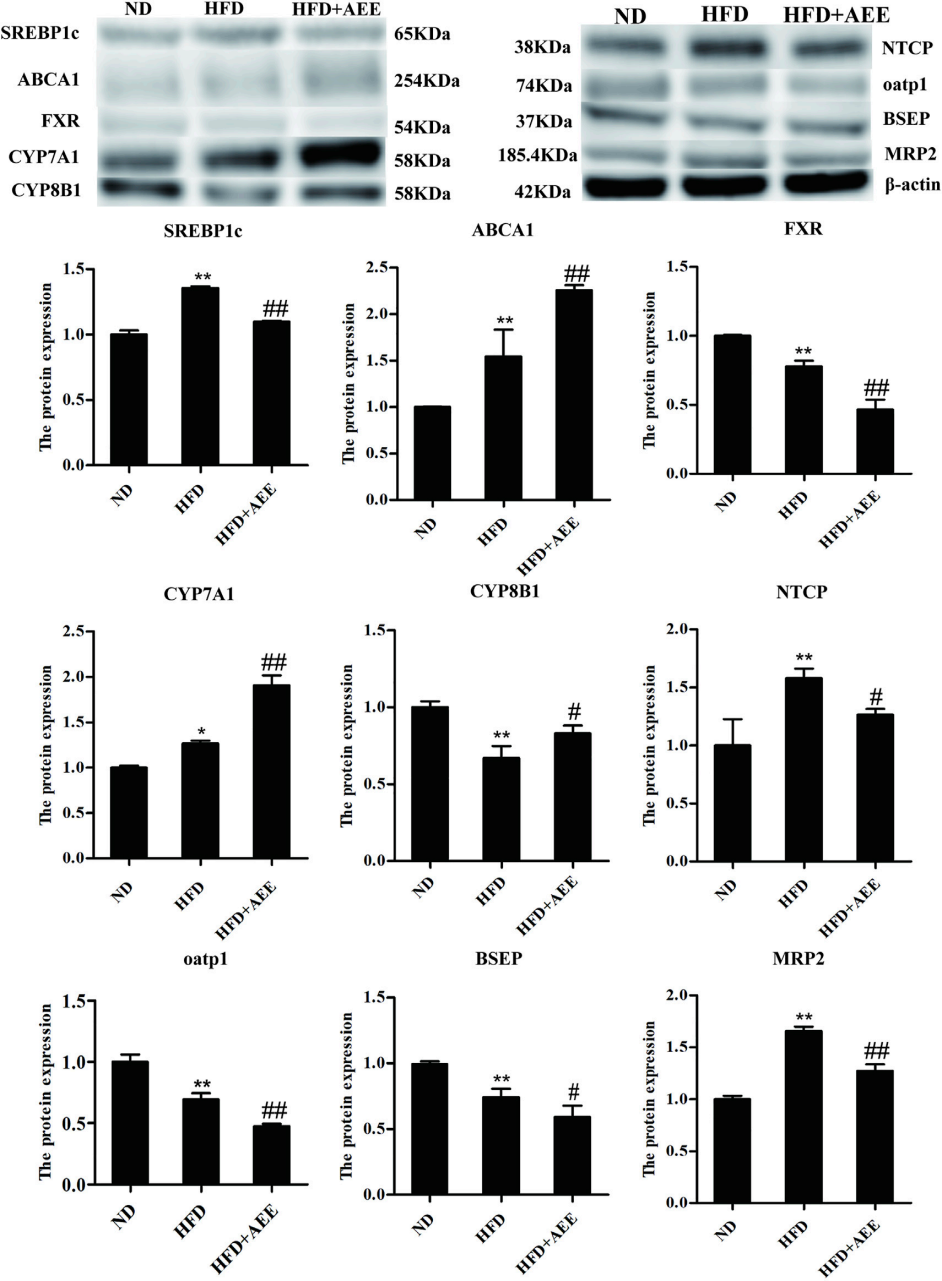
Effects of AEE on protein expression related with cholesterol and BA metabolism in hyperlipidemia rat. Data are expressed as the means ± SD (*n* = 10). HFD, high-fat diet; AEE, aspirin eugenol ester. ^*^ *p* < 0.05, ^**^ *p* < 0.01 compared with the ND group; ^#^ *p* < 0.05, ^##^ *p* < 0.01 compared with the HFD group.

As shown in Figure 6, similar to the results of RT-qPCR, the western blot analysis showed that HFD-mediated markedly increased the protein levels of SREBP1c, ABCA1, NTCP, and MRP2, and decreased FXR, CYP8B1, BSEP, and oatp1 protein levels. AEE markedly elevated the protein levels of SREBP1c, ABCA1, and CYP7A1, reduced FXR, MRP2, and oatp1, compared to HFD group. Meanwhile, AEE treatment elevated the protein level of CYP8B1, and reduced NTCP and BSEP. All the uncropped immunoblotting images of the proteins were shown in Supplementary Figure S8.

### BA Composition in the Liver

The levels of AlloCA, UDCA, and MoCA were unchanged in all groups. Meanwhile, AEE increased the G-BA content and decreased the T-BA content.

**FIGURE 7:**
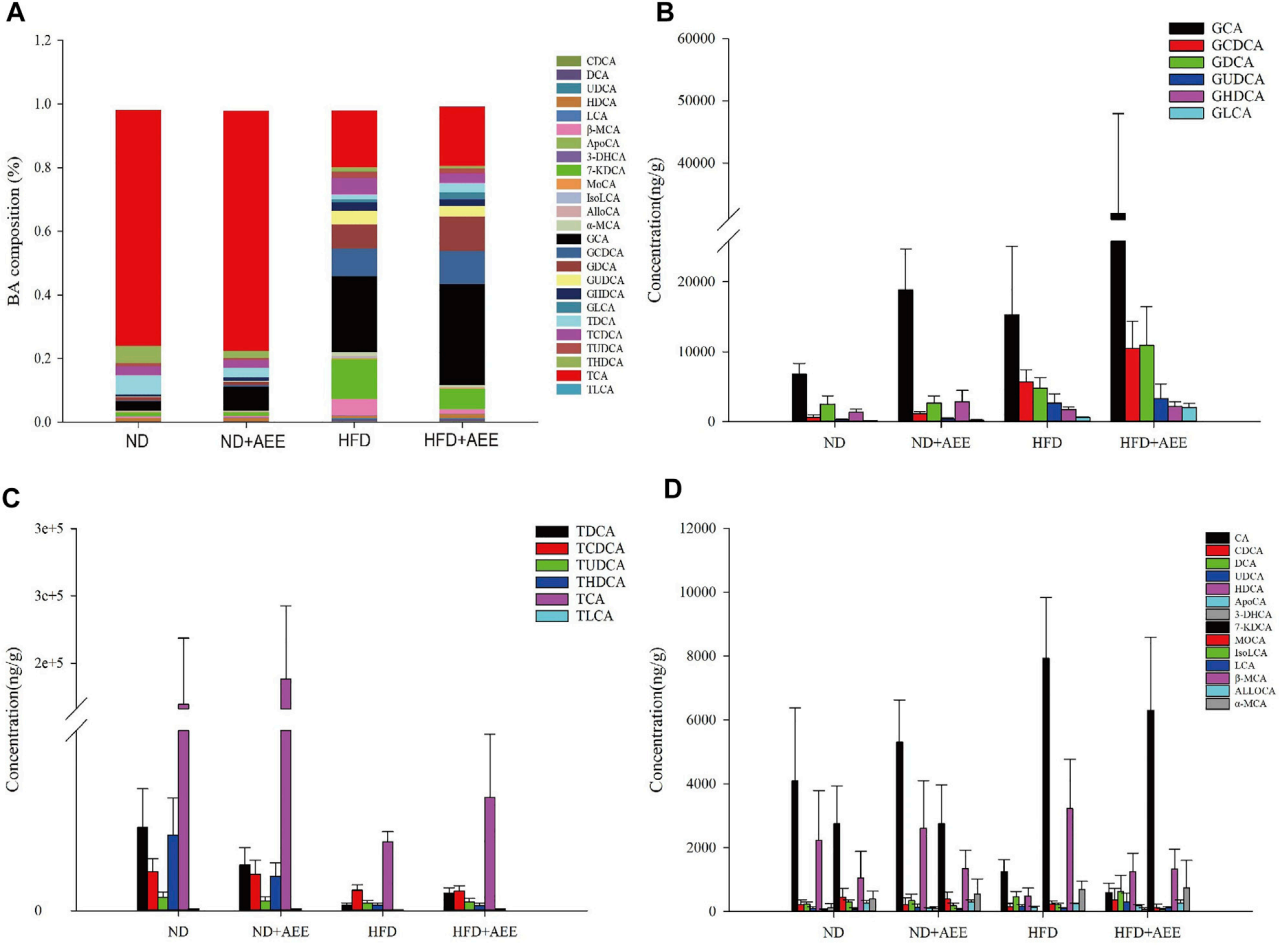
AEE normalized the dysbiosis of BAs composition in hyperlipidemia rats induced by HFD. **(A)** Liver BA profiles are changed after HFD-mediated and AEE treatment. **(B)** The changes of G-BA contents in ND, ND + AEE, HFD, and HFD + AEE group. **(C)** The changes of T-BA contents in ND, ND + AEE, HFD, and HFD + AEE group. **(D)** The changes of unconjugated BAs in ND, ND + AEE, HFD, and HFD + AEE group. ND, control diet; HFD, high-fat diet; AEE, aspirin eugenol ester.

As shown in Figure 7A, the greatest content was TCA in the groups of ND and ND + AEE. However, GCA was the greatest content in the groups of HFD and HFD + AEE. TCA was decreased by 15 times in HFD group in comparison with ND group. Interestingly, an elevation tendency of TCA by AEE treatment was observed in HFD + AEE group. GCA was the major component of G-BAs and increased by three times in ND + AEE group and two times in HFD group compared to ND group, and increased by two times in HFD + AEE group compared to HFD group.

As shown in Figures 7B–D, in ND and ND + AEE group, the results revealed that the levels of G-BAs and ApoCA were dramatically elevated and the T-BAs and IsoLCA were significantly decreased after AEE treatment. The bile acids with elevation tendency were CA, β-MCA, TCA, DCA, HDCA, and GDCA and the levels with decrease tendency were TCDCA, TLCA, TUDCA,7-KDCA, and 3-DHCA in the ND + AEE group, compared to ND group. In ND and HFD group, the results showed that the levels of glycine-conjugated BAs, β-MCA, DCA, KDCA, and ApoCA were dramatically elevated and the tauro-conjugated BAs, CA, HDCA, IsoLCA, and 3-DHCA were significantly decreased after HFD induced. Furthermore, GHDCA had an elevation tendency. In HFD and HFD + AEE group, the results revealed that the levels of glycine-conjugated BAs, HDCA, TDCA, TLCA, ApoCA, and 3-DHCA were dramatically elevated and IsoLCA, CA, and IsoLCA were significantly decreased after AEE treatment. The bile acids with elevation tendency were GHDCA and GUDCA, and the levels with decreased tendency were tauro-conjugated BAs, DCA, and 7-KDCA in the HFD + AEE group, compared to HFD group.

## Discussion

In present study, we investigated the underlying mechanism of AEE on hyperlipidemia. Basing on the approach of untargeted metabolomics using UPLC-Q-TOF/MS, we investigated the effects of AEE on liver and feces in hyperlipidemia hamster. The findings showed that action mechanism of AEE on hyperlipidemia might be mainly involved in regulating amino acid metabolism, glutathione metabolism, energy metabolism, bile acid metabolism, and glycerophospholipid metabolism. A growing body of research showed that BAs and cholesterol metabolisms played an important role in hyperlipidemia. Hence, to gain further insight into the underlying mechanism of AEE on hyperlipidemia, the expression of genes and proteins related to cholesterol and BA metabolism in hyperlipidemia rat were performed. It was found that AEE inhibited FXR expression and upregulated CYP7A1, which was beneficial for hyperlipidemia treatment through facilitating the conversion of cholesterol into BAs. To analyze whether the content of BAs was changed in AEE treatment of hyperlipidemia, the targeted metabolomic analysis of 26 liver BAs was performed by the approach of LC-MS/MS. The content of liver BAs fluctuated with AEE treatment, especially the elevating levels of G-BAs and decreasing levels of T-BAs. Therefore, taken together, these results revealed that AEE ameliorated hyperlipidemia *via* regulating liver and fecal metabolomics profiles, FXR-mediated upregulating of CYP7A1, and upregulating G-BA levels and downregulating T-BA levels.

Hypercholesterolemia, hypertriglyceridemia, and combined hyperlipidemia are different types of hyperlipidemias. Usually, HFD can induce the combined hyperlipidemia in rats and mice with increasing levels of TG, TC, and LDL and/or decreasing level of HDL. Studies showed that the blood lipid disorder, the abnormal levels of TG, TCH, LDL, and HDL, is one of the main features of hyperlipidemia (Wasan et al., 1997; Li et al., 2016a). In our previous studies, we found that AEE could improve hyperlipidemia in rats *via* decreasing the blood lipid levels of TG, TC, and LDL (Karam et al., 2015; Karam et al., 2016). Similar to our previous results, the improvement effects of AEE on blood lipids were observed in this study.

In this study, liver and feces samples from hamster fed with HFD were analyzed using an UPLC-Q-TOF/MS-based metabolomic approach. AEE was administered to investigate its intervention effects in the liver and feces. The PLS-DA score plots indicated that liver and feces metabolic signatures of the control, model, and AEE groups were different. AEE substantially reversed the alterations in the hamster with hyperlipidemia. Moreover, 28 metabolites were selected as potential biomarkers, and its biological function and relevant pathway were identified to elucidate the underlying mechanism of AEE. In our previous study, with the application of hyperlipidemic rats, some potential biomarkers in liver and feces associated with therapeutic effects of AEE were identified (Ma et al., 2017). Interestingly, it was found that these biomarkers were mainly mapped to the metabolisms of glycerophospholipid, amino acid, bile acid, and energy metabolism, which were consistent with the results in present study. When compared with the relative abundance of metabolites found in hyperlipidemic rats, different changing trends of some metabolites such as valine, LysoPC (18:0), phenylalanine, and phytosphingosine were found in liver and feces from hamster with hyperlipidemia. These discrepancies might be caused by the different animal models and different dosage and administration time of AEE.

In the liver of hamster, some metabolites related to amino acid metabolism showed perturbations, including methionine, valine, phenylalanine, tyrosine, and tryptophan. Liver is the major organ involved in amino acid metabolism and largely responsible for maintaining amino acid homeostasis. Compared with the control, the amino acid levels were significantly reduced in hamster, which matched the other reports that amino acid levels had dropped by half in the liver of obese mice after 12 weeks of HFD feeding (Park et al., 2017). Importantly, amino acid levels were increased through AEE treatment, indicating AEE might normalize abnormal amino acid metabolisms generated by HFD. Therefore, it was assumed that the anti-hyperlipidemia efficacy of AEE might ascribe to the promotion of amino acid metabolism and restored protein synthesis, which were predominantly associated with oxidation, inflammation, and lipid metabolism. For example, methionine plays key roles in lipid metabolism, oxidative stress, and bile metabolism in hepatocytes, and tryptophan can effectively alleviate hepatitis through reducing the proinflammatory cytokines levels (Celinski et al., 2014). Valine as an antioxidant can improve the endothelial dysfunctions through reducing the levels of reactive oxygen species (Martin-Lorenzo et al., 2016). So the increased levels of methionine, valine, and tryptophan caused by AEE might improve lipid and bile metabolism, and reduce oxidative stress and inflammation, which were all beneficial for hyperlipidemia treatment.

Glutathione metabolism plays important roles in antioxidant defense, free radicals neutralization, and NO regulation, which has been demonstrated to be a major mechanism involved in the initiation and progression of hyperlipidemia (Wu et al., 2004). Pyroglutamic acid is a metabolite in the glutathione cycle. Glutathione and pyroglutamic acid are indicators of endogenous antioxidative system. Oxidative stress is widely described to be an important factor in atherosclerosis. The reduction of pyroglutamic acid and the increase of glutathione in the model group suggested that hamster fed with HFD suffered from oxidative stress (Jiang et al., 2013). In contrast to the healthy rats, increased glutathione in the liver of the hamster suggested that more antioxidants were produced to defend the increasing oxidative stress during pathological progression. AEE might improve the abnormality of glutathione and pyroglutamic acid through ameliorating the oxidative stress induced by HFD.

Glycerophospholipid metabolism plays key roles in platelet aggregation, inflammatory diseases, and hyperlipidemia development. LysoPCs are formed by hydrolysis of phosphatidylcholines, and its increase can trigger inflammation and the autoimmune response in atherosclerosis (Li et al., 2016b). In this study, LysoPC (18:0), LysoPC (16:1), and LysoPC (20:3) were increased in the liver of the hamster, which were consistent with previous studies (Liu et al., 2014). AEE treatment showed favorable inhibition of these increased LysoPCs, implying the inhibitory effects of AEE on glycerophospholipid metabolism. Inflammation was a key part in the pathogenesis of atherosclerosis. Low levels of LysoPCs in the liver caused by AEE treatment were conducive to reducing inflammation. Therefore, AEE played a key role in regulating the disorders of glycerophospholipid metabolism, which could reduce inflammation and inhibit the progress of hyperlipidemia.

Palmitic acid and stearic acid are common saturated fatty acid. Previous studies demonstrated that the fatty acid synthesis is generally increased, and then lead the elevation of palmitic acid which could promote progression of steatosis to steatohepatitis in HFD-fed mice (Kwan et al., 2015). Moreover, palmitic acid can activate the inflammatory process, and promote cholesterol accumulation in LDL particles and macrophages (Afonso et al., 2016). Interestingly, our results indicated that the level of palmitic acid was significant higher in the liver of the model group when compared with the control, indicating the promotion in fatty acid metabolism. The increased palmitic acid was found to be down regulated by AEE treatment, which was beneficial for hyperlipidemia treatment through reducing cholesterol accumulation and inflammation inhibition. Craig et al. reported that dietary stearic acid could reduce plasma cholesterol concentration in hamsters, which might be caused by reduced cholesterol absorption and increased excretion of endogenous cholesterol (Schneider et al., 2000). Decreased stearic acid caused by HFD in the liver of hamster was reversed by AEE treatment, which could lead to the reduction of cholesterol. In addition, it was suggested that an increased ratio of palmitic acid to stearic acid could be considered as an indicator for fatty acid metabolism disorder (Zhao et al., 2017). Increased ratio of palmitic acid to stearic acid was inhibited by AEE treatment through the upregulation of stearic acid and downregulation of palmitic acid, suggesting that AEE could ameliorate the disturbed fatty acid metabolism.

Malic acid is an intermediate in krebs cycle. Decreasing of malic acid in model group indicated that the energy production from krebs cycle was inhibited (Mayr et al., 2005). In this study, decreased levels of malic acid might imply that the krebs cycle was inhibited in response to hyperlipidemia. AEE treatment corrected the alternation of malic acid, suggesting that AEE could regulate the dysfunction of energy metabolism.

Bile acids can facilitate the absorption, transport, and excretion of sterols and fats in the liver and intestine. Cholic acid is a major primary bile acid produced in the liver. Glycoursodeoxycholic acid (GUDCA) and glycocholic acid are secondary bile acids produced by the action of enzymes existing in the colonic microbial flora. The increased levels of GUDCA in liver of hamster were observed, which was in accordance with the reported results (Liu et al., 2014). AEE inhibited the upregulation of GUDCA, suggesting that AEE could ameliorate the disturbed bile acid metabolism. Hamsters fed with HFD showed elevated levels of cholic acid and glycocholic acid, which indicated the promotion of bile acid synthesis. Compared with the model group, a sharp increase of cholic acid and glycocholic acid in feces were observed. It has been reported that increased levels of bile acids are contributed to eliminating cholesterol, which is essential for inhibiting atherosclerosis formation (Liu et al., 2017). Therefore, it was speculated that AEE could contribute to excreting cholesterol through increased bile acids in feces, and thus inhibit the development and progression of hyperlipidemia. It was reported that phytosphingosine was increased in the plasma of the atherosclerosis rabbit, which may inhibit the reverse cholesterol transport pathway, resulting in the increase risk of atherosclerosis (Liu et al., 2014). Increased level of phytosphingosine in the feces was observed in the atherosclerosis hamster. After AEE treatment, phytosphingosine was downregulated in the feces, indicating the regulation effects of AEE on cholesterol transport.

Bile acids are endogenous signaling molecules that bind to the BA receptors, FXR and G protein-coupled receptor (TGR5), regulating BA homeostasis in the enterohepatic circulation, modulating cholesterol and triglyceride metabolism, and maintaining glucose and energy homeostasis (Kuipers et al., 2014; Liu and Wang, 2019). The synthesis of BA is regulated *via* feedback mechanism mediated by CYP7A1 (Russell and Setchell, 1992). FXR regulates hepatic BA biosynthesis, transport, and secretion. Sirtuin 1 (Sirt1), a class Ⅲ NAD+-dependent histone deacetylase, regulates lipid, glucose, and bile acid metabolism (Yang et al., 2017a). Studies showed that Sirt1 is critical for liver function and is pivotal to improve hyperlipidemia and hepatic lipid metabolism (Martins, 2017). The orphan nuclear receptor FXR is the master regulator of BA, lipid, and glucose metabolism. Sirt1 directly modulates FXR activity by deacetylation of this nuclear receptor and neighboring histones that strictly control target gene transcription. Hepatic Sirt1 is a key regulator of the FXR signaling pathway and hepatic metabolism homeostasis (Garcia-Rodriguez et al., 2014; Sun et al., 2020). Hence, Sirt1 may be a potential target of AEE. Further studies are needed to explore the effects of AEE on Sirt1, which is important to understand the action mechanism of AEE on hyperlipidemia.

In the liver, FXR inhibits CYP8B1 expression and bile acid synthesis (Hu et al., 2014). Numerous studies indicated that FXR inhibits CYP7A1 which is a rate-limiting enzyme of hepatic bile acids synthesis (Thomas et al., 2008; Jia et al., 2018). In this study, AEE treatment downregulated the expression of FXR and upregulated CYP7A1 and CYP8B1 at the both levels of mRNA and protein. Hence, the increased expression of CYP7A1 and CYP8B1 could accelerate the conversion of cholesterol into bile acid in liver, which was induced by decrease expression of FXR in liver by AEE treatment. Fecal TBA excretion was increased noticeably in AEE treated rats. Hence, AEE might promote the production of hepatic BAs *via* feedback mechanism by CYP7A1, resulting increased fecal TBA excretion, which could make contribution to the improvement effects of AEE on hyperlipidemia.

In addition, AEE treatment was shown to markedly decrease the mRNA and protein levels of transporters regulating the BA reabsorption and exportation in the BAs enterohepatic circulation, NTCP, OATP1, BSEP, and MRP2 in the rat liver. Therefore, it was conjectured that AEE decreased the content of BAs in rats. Research suggested that HFD-fed BSEP^+/−^ mice exhibited milder hepatic steatosis and less weight gain compared to HFD-fed wild-type mice (Okushin et al., 2020). Hence, the decreased level of BSEP is likely a mechanism for AEE to achieved the purpose of improving hyperlipidemia. Studies also reported that enhanced fecal BA loss is accompanied by enhanced hepatic BA synthesis, which is a key pathway to relieve cholesterol related diseases (Charach et al., 2011; Charach et al., 2018). Therefore, it was speculated that AEE decreased the content of BAs in rat *via* excreting in feces and achieved the purpose of improving hyperlipidemia.

In hepatocytes, cholesterol is converted into primary BAs by 17 enzymes and then most BAs are combined with glycine or taurine to form conjugated bile acids (Yang et al., 2017b; Jia et al., 2018; Kirilenko et al., 2019). In this study, the results showed that HFD-mediated significantly decreased the content of TCA and increased the content of GCA, which were the important part of T-BAs and G-BAs, respectively. TCA level was an elevation tendency by AEE treatment, but other T-BA levels acquired a depressed tendency. However, the content of GCA obviously increased after AEE administration and other G-BA levels got an increase tendency. Therefore, the development of hyperlipidemia is accompanied by the fluctuate of G-BAs and T-BAs in the liver, which may be an important part of the improvement of hyperlipidemia by AEE administration. In comparison with HFD group, the level of HDCA increased significantly and the β-MCA level decreased in HFD + AEE group. Previous study showed that HDCA altered BA metabolism profiles induced by gut bacteria (Song et al., 2020). Meanwhile, it was found that HDCA was a candidate for anti-atherosclerotic drug therapy *via* increasing the ability of HDL (Shih et al., 2013). In present study, reduced level of HDCA caused by AEE treatment might change the gut bacteria and HDL ability, which was beneficial for hyperlipidemia treatment. AEE treatment significantly decreased the level of IsoLCA and increased the level of ApoCA. CA was an activator of CYP7A1, and reduction in circulation CA increased CYP7A1 activation (Li-Hawkins et al., 2002). AEE treatment decreased the content of CA and significantly increased the CYP7A1 expression, which was consistent with the early studies. CDCA, LCA, DCA, and CA are FXR promoters (Parks et al., 1999).

## Conclusion

The untargeted metabolomics result showed that AEE could regulate the metabolic disorders in liver and feces induced by hyperlipidemia. Twenty-four liver metabolites and four fecal metabolites were selected and identified in response to hyperlipidemia, and AEE normalized these metabolite alternations. Pathway analysis results suggested that effects of AEE on hyperlipidemia were related with the regulating of amino acid metabolism, glutathione metabolism, energy metabolism, bile acid metabolism, and glycerophospholipid metabolism. AEE reduced cholesterol accumulation by FXR-mediated upregulating of CYP7A1, hepatic BA synthetic gene, and increased fecal BA excretion. The effect of AEE on hyperlipidemia is accompanied by the improvement of BA content in the liver, especially the increase of G-BAs and decrease of T-BAs. The study provided new evidence for the possible molecular mechanisms and targets of AEE for anti-hyperlipidemia therapies. It was also demonstrated that the combination of untargeted metabonomic and targeted metabonomic approach was a powerful tool in investigating drug action mechanism.

## Data Availability

The original contributions presented in the study are included in the article/Supplementary Materials, further inquiries can be directed to the corresponding authors.
