# Supplementary material for: Untargeted and Targeted Metabolomics Reveal the Underlying Mechanism of Aspirin Eugenol Ester Ameliorating Rat Hyperlipidemia via Inhibiting FXR to Induce CYP7A1
Source: Front Pharmacol. 2021 Nov 25;12:733789. doi: 10.3389/fphar.2021.733789 (PMC8656224; doi:10.3389/fphar.2021.733789)
Supplement: Supplementary file 2 [file DataSheet2.docx]

**Fig. S1** Study design of the untargeted metabolomic experiment.


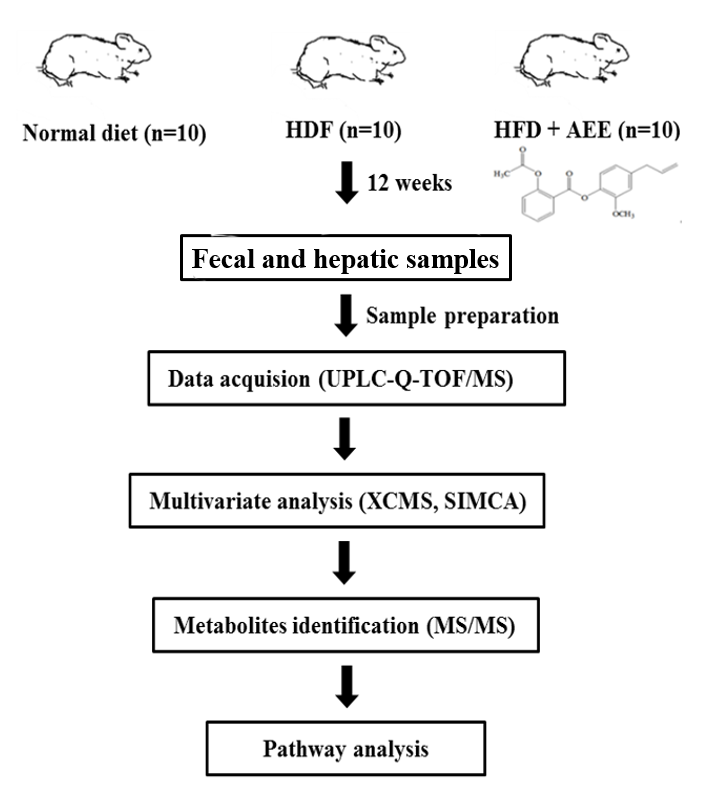


**Figure S2** Effect of AEE on liver histology in hyperlipidemia rat.


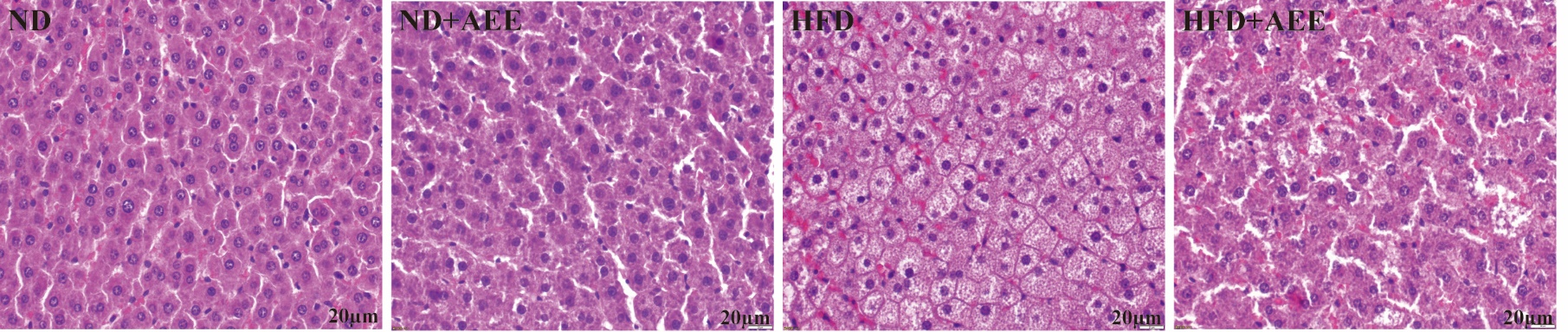


Histopathological H&E staining of rat liver tissue after AEE treatment (scale bar = 100µm). ND: Normal diet; HFD: high-fat diet; AEE: aspirin eugenol ester. In comparison with ND group, there was no pathological change in the liver of the ND+AEE group, but the fatty degenerations and vacuoles of liver cells in HFD group were obvious. AEE treatment significantly improved the pathological changes

**Figure S3** Typical UPLC-Q-TOF/MS total ion chromatograms of hamster liver in positive and negative ion modes.

ESI+

| ESI- 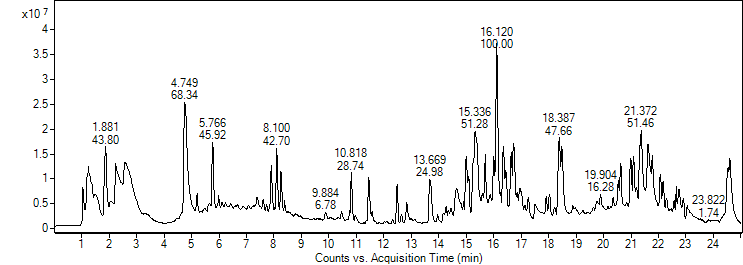 |
| --- |
| 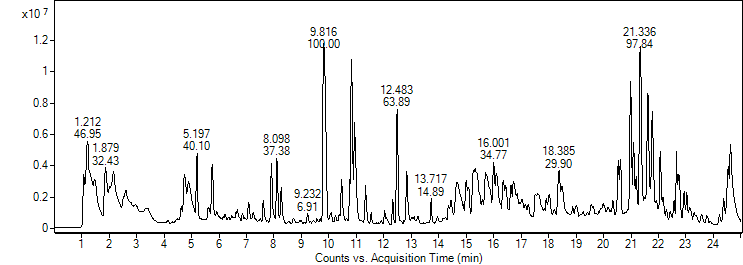 |

The retention time and relative intensity were labeled on the peaks.

**Figure S4** PCA score plots of liver analyzed by UPLC-TOF/MS in positive and negative modes.


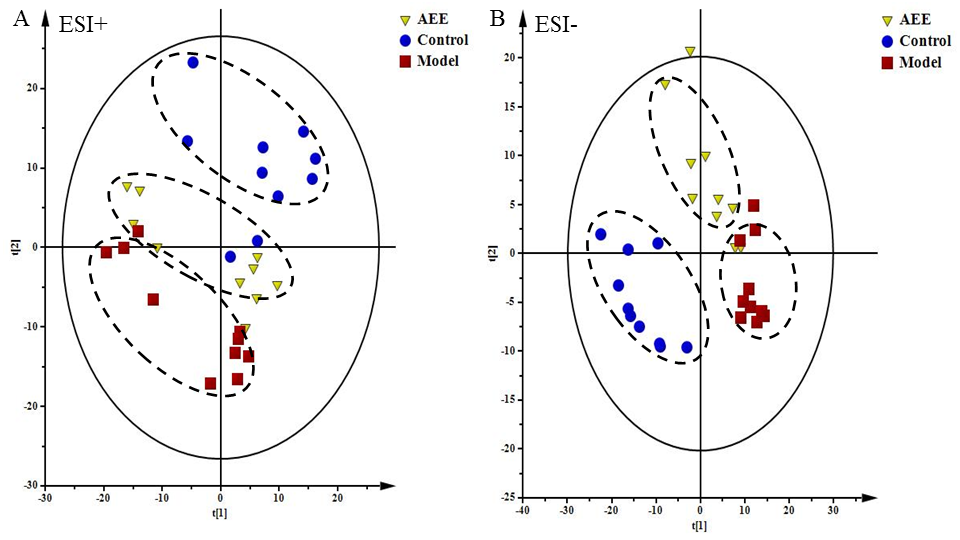


PCA score plots of control, model and AEE groups. ESI+: R^2^X = 0.416, ESI-: R^2^X = 0.406 (n = 10 in each group).

**Figure S5** Typical UPLC-Q-TOF/MS total ion chromatograms of hamster feces in positive and negative ion modes.


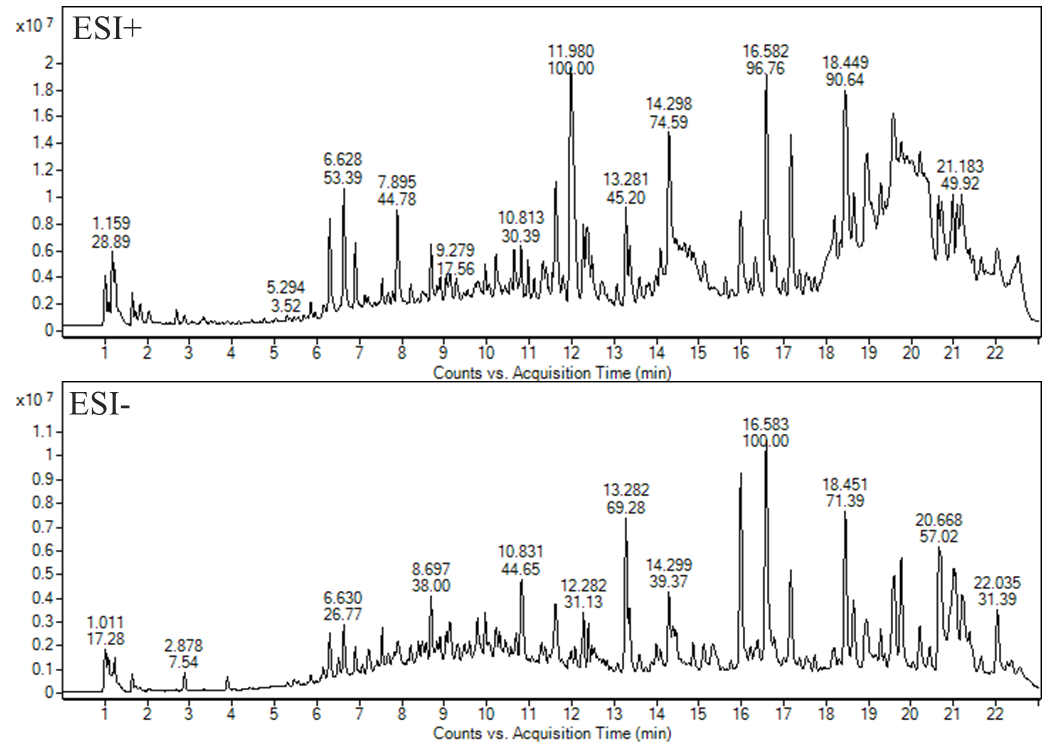


The retention time and relative intensity were labeled on the peaks.

.

**Figure S6** PCA score plots of feces analyzed by UPLC-TOF/MS in the positive and negative modes.


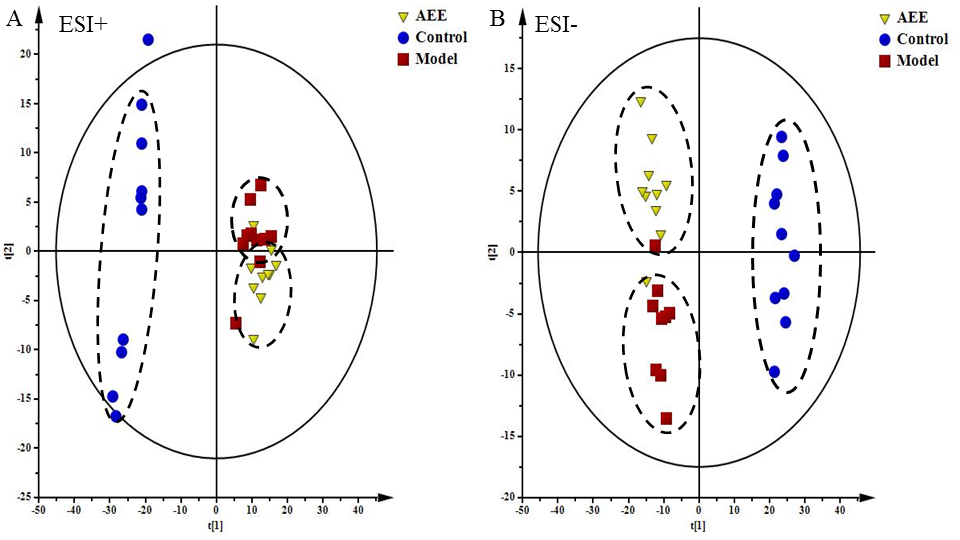


PCA score plots of control, model and AEE groups. ESI+: R^2^X = 0.463, ESI-: R^2^X = 0.512. (ESI+: n = 10 in each group; ESI-: n = 10 in control and AEE groups, n = 9 in model group that an outlier was far outside the ellipse representing 95% confidence interval

**Fig. S7** Heatmap and cluster analysis of the significantly changed metabolites in different groups.


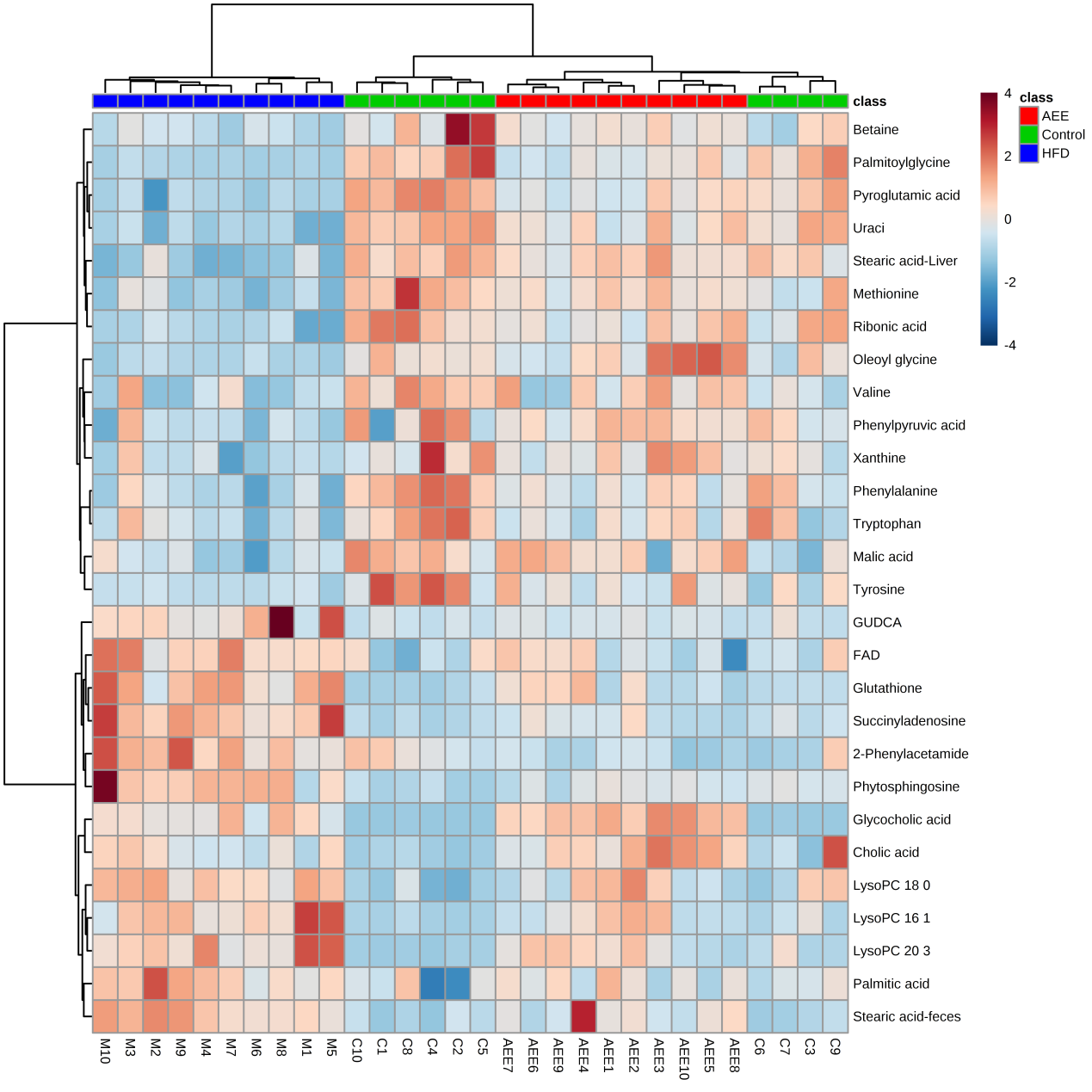


C: samples in control group; M: samples in HFD group; AEE: samples in AEE group

**Figure S8** All the uncropped immunoblotting images of the proteins.


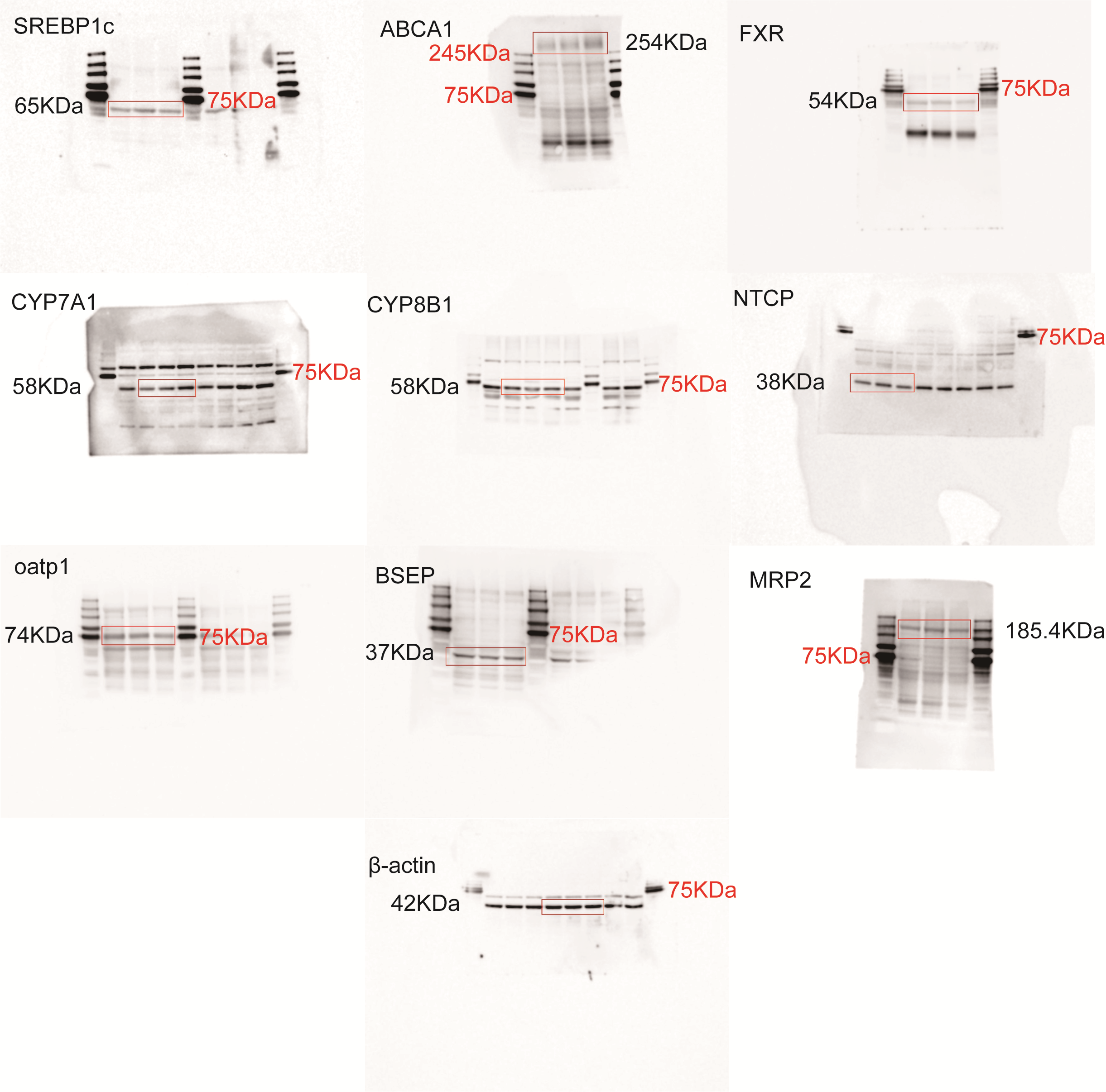


The typical protein signals of the liver samples were labeled in the red box, and the molecular weight of the proteins was marked in the figure.

**Table S1** UPLC gradient elution program.

| Sample | Time (min) | A% | B% |
| --- | --- | --- | --- |
| Feces | 0 | 95 | 5 |
|  | 3 | 80 | 20 |
|  | 8 | 60 | 40 |
|  | 10 | 40 | 60 |
|  | 20 | 5 | 95 |
|  | 21 | 5 | 95 |
|  | 22 | 95 | 5 |
|  | 23 | 95 | 5 |
| Liver | 0 | 98 | 2 |
|  | 2 | 98 | 2 |
|  | 9 | 55 | 45 |
|  | 15 | 30 | 70 |
|  | 22 | 2 | 98 |
|  | 23 | 2 | 98 |
|  | 24 | 98 | 2 |
|  | 25 | 98 | 2 |

Mobile phase A: water with 0.1% formic acid; Mobile phase B: acetonitrile with 0.1% formic acid (B). Flow rates of both stool and liver samples were 0.3 mL/min.

**Table S2** Primers used for gene expression analysis.

| Gene | Forward Primer (5´→3´) | Reverse Primer (3´→5´) |
| --- | --- | --- |
| SREBP1c | CAGCAGCAGTGGTGGCAGTG (20) | GGTTGCAGGTCAGACACAGGAAG (23) |
| ABCA1 | TCAATGAGACCAACCAGGCAATCC (24) | ACTTGTTGATGAGCGTGACTTCGG (24) |
| CYP7A1 | GCAGCCTCTGAAGAAGTGAGTGG (23) | AGCTGTGCGGATATTCAAGGATGC (24) |
| FXR | CGTCGGAAGTGCCAGGATTGC (21) | CCTTCGCTGTCCTCATTCACTGTC (24) |
| CYP8B1 | GCGATGAAGGCTGTGCGAGAG (21) | GTCTCTTCCATCACGCTGTCCAG (23) |
| NTCP | TCCTCATCTGTGGCTGCTCTCC (22) | ATCACGATGCTGAGGTTCATGTCC (24) |
| oatp1 | GGATCAAGCTATCTGCCTGCCTTC (24) | GACCTGAGGACTTCCATGCACATC (24) |
| BSEP | CCGTTCTGTTCTCCACCACTATCG (24) | TTGCTTCTGACCACCACTCATCTG (24) |
| MRP2 | GGTCGTCTTCTGTTCCGCCTTG (22) | ACTGCCACAATGTTGGTCTCTGC (23) |
| β-actin | GGCCAACCGTGAAAAGATGA (20) | CAGCCTGGATGGCTACGTACA (21) |

**Table S3** Antibodies used for protein expression analysis.

| Name | Source | Lot | dilution |
| --- | --- | --- | --- |
| SREBP1c | Abcam, UK | ab28481 | 1:1000 |
| ABCA1 | Abcam, UK | ab18180 | 1:300 |
| FXR | Invitrogen, US | 417200 | 1:1000 |
| CYP7A1 | Abcam, UK | ab65596 | 1:1000 |
| CYP8B1 | Abcam, UK | ab191910 | 1:1000 |
| NTCP | Abcam, UK | ab131084 | 1:1000 |
| oatp1 | Abcam, UK | ab203036 | 1:500 |
| BSEP | Invitrogen, US | PA5-13105 | 1:1000 |
| MRP2 | Abcam, UK | ab15603 | 1:30 |
| β-actin | Abcam, UK | Ab8227 | 1:5000 |
| Mouse secondary antibody | Abcam, UK | ab6789 | 1:2000 |
| Goat secondary antibody | Abcam, UK | ab6721 | 1:2000 |

**Table S4** The 26 kinds of liver BAs were quantified by LC-MS/MS.

| Component Name | Mass Information | Retention Time (min) | Linear | R^2^ |
| --- | --- | --- | --- | --- |
| Cholic acid (CA) | 407.4 / 407.4 | 8.50 | Y = 0.00884 X - 0.01874 | 0.99988 |
| Chenodeoxycholic acid (CDCA) | 391.4 / 391.4 | 11.70 | Y = 0.02012 X + 0.05158 | 0.99990 |
| Deoxycholic acid (DCA) | 391.4 / 391.4 | 12.08 | Y = 0.02827 X + 0.02349 | 0.99978 |
| Ursodeoxycholic acid (UDCA) | 391.4 / 391.4 | 6.87 | Y = 0.02169 X + 0.01773 | 0.99991 |
| Hyodeoxycholic acid (HDCA) | 391.4 / 391.4 | 7.97 | Y = 0.01485 X + 0.04444 | 0.99982 |
| Glycocholic acid (GCA) | 464.4 / 74.0 | 5.78 | Y = 0.01439 X - 0.04576 | 0.99937 |
| Glycochenodeoxycholic acid (GCDCA) | 448.4 / 74.0 | 8.42 | Y = 0.05002 X-0.02544 | 0.99978 |
| Glycodeoxycholic acid (GDCA) | 448.4 / 73.9 | 9.21 | Y = 0.04098 X-0.15408 | 0.99979 |
| Glycoursodeoxycholic acid (GUDCA) | 448.4 / 73.9 | 4.09 | Y = 0.01121 X - 0.01139 | 0.99985 |
| Glycohyodeoxycholic acid (GHDCA) | 448.4 / 74.1 | 4.64 | Y = 0.01227 X - 0.02089 | 0.99984 |
| Taurocodeoxycholic acid (TDCA) | 498.4 / 80.0 | 6.30 | Y = 0.03413 X + 0.40549 | 0.99991 |
| Taurochenodeoxycholic acid (TCDCA) | 498.4 / 80.0 | 5.70 | Y = 0.00645 X-0.00277 | 0.99915 |
| Tauroursodeoxycholic acid (TUDCA) | 498.4 / 79.8 | 2.62 | Y = 0.02396 X - 0.02241 | 0.99882 |
| Taurohyodeoxycholic acid (THDCA) | 498.4 / 79.9 | 2.90 | Y = 0.03319 X - 0.0594 | 0.99963 |
| Glycolithocholic acid (GLCA) | 432.5 / 73.9 | 11.59 | Y = 0.01804 X - 0.00736 | 0.99995 |
| Lithocholic acid (LCA) | 375.3 / 375.3 | 14.59 | Y = 0.00579 X + 0.00238 | 0.99963 |
| Taurocholic acid (TCA) | 514.4 / 79.9 | 3.69 | Y = 0.00923 X + 0.10422 | 0.99870 |
| Taurolithocholic acid (TLCA) | 482.4 / 80.0 | 8.57 | Y = 0.0238 X + 0.03825 | 0.99986 |
| α-Muricholic acid (α-MCA) | 407.4 / 407.4 | 5.07 | Y = 0.04086 X+0.10141 | 0.99934 |
| β-Muricholic acid (β-MCA) | 407.4 / 407.4 | 5.39 | Y = 0.02081 X + 0.05081 | 0.99993 |
| Apocholic acid (ApoCA) | 389.4 / 389.4 | 10.24 | Y = 0.02337 X + 0.00153 | 0.99918 |
| 3-dehydrocholic acid/3-oxocholic acid (3-DHCA) | 405.4 / 405.4 | 6.34 | Y = 0.0234 X + 0.00437 | 0.99956 |
| 7-ketodeoxycholic acid (7-KDCA) | 405.4 / 405.4 | 4.80 | Y = 0.00123 X - 0.00187 | 0.99888 |
| murocholic acid (MoCA) | 391.4 / 391.4 | 5.77 | Y = 0.0179 X + 0.01132 | 0.99982 |
| Isolithocholic acid (IsoLCA) | 375.5 / 375.5 | 12.67 | Y = 0.00184 X + 0.0013 | 0.99758 |
| Allocholic acid (AlloCA) | 407.4 / 361.2 | 8.69 | Y = 0.0023 X - 0.00514 | 0.99982 |

**Table S5** Pathway analysis results from MetaboAnalyst.

| Pathway name | Total | Expected | Hits | Raw p | -log (p) | Holm adjust | FDR | Impact |
| --- | --- | --- | --- | --- | --- | --- | --- | --- |
| Phenylalanine, tyrosine and tryptophan biosynthesis | 4 | 0.059914 | 3 | 1.15E-05 | 11.374 | 0.00091966 | 0.000466 | 1 |
| Phenylalanine metabolism | 9 | 0.13481 | 4 | 4.48E-06 | 12.316 | 0.00036288 | 0.000363 | 0.77777 |
| Ubiquinone and other terpenoid-quinone biosynthesis | 3 | 0.044936 | 1 | 0.044297 | 3.1168 | 1 | 0.71762 | 0.5 |
| Glutathione metabolism | 26 | 0.38944 | 2 | 0.055965 | 2.883 | 1 | 0.75552 | 0.17647 |
| Valine, leucine and isoleucine biosynthesis | 11 | 0.16476 | 1 | 0.15347 | 1.8742 | 1 | 1 | 0.14286 |
| Tryptophan metabolism | 41 | 0.61412 | 1 | 0.46626 | 0.76302 | 1 | 1 | 0.12821 |
| Tyrosine metabolism | 42 | 0.6291 | 1 | 0.47449 | 0.74551 | 1 | 1 | 0.11905 |
| Aminoacyl-tRNA biosynthesis | 67 | 1.0036 | 5 | 0.002405 | 6.0301 | 0.19002 | 0.064944 | 0.10415 |
| Riboflavin metabolism | 11 | 0.16476 | 1 | 0.15347 | 1.8742 | 1 | 1 | 0.1 |
| Glyoxylate and dicarboxylate metabolism | 16 | 0.23966 | 1 | 0.21556 | 1.5345 | 1 | 1 | 0.08333 |
| Pantothenate and CoA biosynthesis | 15 | 0.22468 | 2 | 0.019959 | 3.9141 | 1 | 0.40416 | 0.07692 |
| Pyruvate metabolism | 22 | 0.32953 | 1 | 0.28434 | 1.2576 | 1 | 1 | 0.07407 |
| Citrate cycle (TCA cycle) | 20 | 0.29957 | 1 | 0.26208 | 1.3391 | 1 | 1 | 0.06897 |
| Glycerophospholipid metabolism | 30 | 0.44936 | 1 | 0.36716 | 1.0019 | 1 | 1 | 0.05263 |

Total: The total number of compounds in the pathways; the hits are the actually matched number from the upload data; the raw p is the original p value calculated from the enrichment analysis; the impact is the pathway impact value calculated from pathway analysis.
